# Construction of Ultrathin Layered MXene-TiN Heterostructure Enabling Favorable Catalytic Ability for High-Areal-Capacity Lithium–Sulfur Batteries

**DOI:** 10.1007/s40820-022-00935-0

**Published:** 2022-09-17

**Authors:** Hao Wang, Zhe Cui, Shu-Ang He, Jinqi Zhu, Wei Luo, Qian Liu, Rujia Zou

**Affiliations:** 1 State Key Laboratory for Modification of Chemical Fibers and Polymer Materials, College of Materials Science and Engineering, Donghua University, Shanghai, 201620 People’s Republic of China; 2 Department of Physics, College of Science, Donghua University, Shanghai, 201620 People’s Republic of China

**Keywords:** Li–S batteries, Ultrathin 2D structures, Electrochemical catalysis, MXenes, Ti_3_C_2_T_*x*_-TiN

## Abstract

Catalysis has been regarded as an effective strategy to mitigate sluggish reaction kinetics and serious shuttle effect of Li–S batteries. Herein, a spherical structure consists of ultrathin layered Ti_3_C_2_T_*x*_-TiN heterostructures (MX-TiN) through in-situ nitridation method is reported. Through controllable nitridation, highly conductive TiN layer grew on the surface and close coupled with interior MXene to form unique 2D heterostructures. The ultrathin heterostructure with only several nanometers in thickness enables outstanding ability to shorten electrons diffusion distance during electrochemical reactions and enlarge active surface with abundant adsorptive and catalytic sites. Moreover, the (001) surface of TiN is dominated by metallic Ti–3*d* states, which ensures fast transmitting electrons from high conductive MX-TiN matrix and thus guarantees efficient catalytic performance. Calculations and experiments demonstrate that polysulfides are strongly immobilized on MX-TiN, meanwhile the bidirectional reaction kinetics are catalytically enhanced by reducing the conversion barrier between liquid LiPSs and solid Li_2_S_2_/Li_2_S. As a result, the S/MX-TiN cathode achieves excellent long-term cyclability with extremely low-capacity fading rate of 0.022% over 1000 cycles and remarkable areal capacity of 8.27 mAh cm^−2^ at high sulfur loading and lean electrolytes.

**Graphical abstract:**

**Supplementary Information:**

The online version contains supplementary material available at 10.1007/s40820-022-00935-0.

## Highlights

- An in-situ strategy to synthesize ultrathin two-dimensional MXene-TiN heterostructures and the ultrathin structure extremely shortens the electrons diffusion distance from catalysts to active sulfur species.
- The heterostructure exhibits superior electronic structures to strongly capture the polysulfides and enhance bidirectional electrocatalytic ability between LiPSs and Li_2_S.
- The advanced cathode achieves an excellent long-term cyclability with an extremely low-capacity fading rate of 0.022% over 1000 cycles and a remarkable areal capacity of 8.27 mAh cm^−2^ at high sulfur loading of 10.16 mg cm^−2^.

**Supplementary Information:**

The online version contains supplementary material available at 10.1007/s40820-022-00935-0.

## Graphical abstract

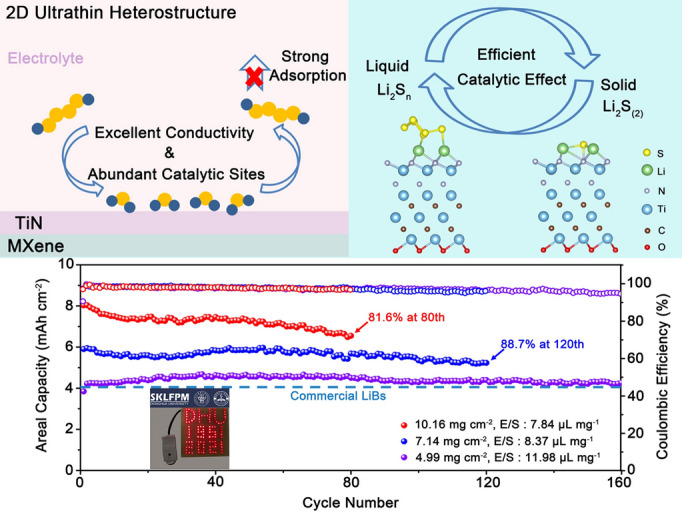

## Introduction

The rapid growing demand for electric vehicles and cheap large-scale energy grids inspire the enthusiasm to explore the next-generation energy storage systems beyond lithium-ion batteries [1–3]. Lithium-sulfur batteries (LSBs) promise significant advantages with respect to high theoretical capacity (1672 mAh g^−1^), prominent energy density (2600 Wh kg^−1^), low cost of sulfur and environmental friendliness [4, 5]. However, the serious shuttle effect caused by soluble intermediate lithium polysulfides (LiPSs) dissolved in liquid electrolytes, the insulating nature of sulfur and lithium sulfide (Li_2_S), and the sluggish sulfur redox kinetics restrict the actual performance of Li–S batteries [6, 7].

In the past decade, most studies have focused on to improve the adsorptive ability to tackle the shuttle effect, including physical confinement (e.g., porous carbon [8] and MOF [9, 10]), and chemical interactions (e.g., metal oxides [11, 12], metal sulfides [13], and metal carbides [14, 15]). However, only adsorption of LiPSs is rather powerless for enhancing electrochemical performance of Li–S batteries because the trapped LiPSs are hard to be effectively converted due to the sluggish sulfur redox kinetics [16]. In addition, researchers found that excessive adsorption will give rise to the decomposition of LiPSs and thus hinder the electrochemical redox reactions [17–19]. Recently, catalytic effect has been recognized as the key technology to improve conversion kinetics and inhibit the shuttle effect simultaneously [20, 21]. The conversion of sulfur species is accelerated and thus the shuttle effect is mitigated [22]. Previous research experiences indicated that the ideal catalysts should possess: (a) moderate adsorption performance to inhibit the shuttle effect; (b) excellent conductivity to promote the electron/ion diffusion kinetics; (c) as well as high specific area with abundant catalytic sites for accelerating LiPSs conversion [23].

MXenes, as a new type of two-dimensional (2D) transition metal carbide/carbonitride materials, are considered as a promising sulfur host material due to their excellent conductivity, high specific surface area, and superior hydrophilicity. However, MXene tends to spontaneously restack to densely film on account of van der Waals forces and hydrogen bonds, which limits the utilization of active surface and reduces the sulfur loading [24]. Moreover, the pure MXene usually represents weak chemical immobilization ability and poor catalytic effect [25]. Heterostructure engineering on MXene is an effective strategy to enhance its adsorptive ability and electrochemical performance. For instance, Yang et al. reported a self-oxidation method to produce TiO_2_-Ti_3_C_2_T_x_ heterostructures, in which the TiO_2_ nanoparticles on MXene sheets act as capturing centers to immobilize LiPSs, and the hetero-interface enhances rapid LiPSs diffusion [26]. Similarly, many other transition metal oxides/sulfides (TMOs/TMSs)-MXene heterostructures have been synthesized to reduce the shuttle effect and improve the cycle stability to some extent [27–29]. However, TMOs/TMSs generally exhibit such poor electrical/ionic conductivity that sluggish sulfur kinetics during redox reactions, which causes serious challenges in attaining high areal capacity Li–S batteries [30, 31]. Even worse, these materials attached on MXene are usually limited to particles or nanoparticles, so that the active heterostructures are deeply embedded inside the bulk and difficult to expose catalytic sites [32]. In order to find efficient and low-cost electrocatalysts, transition metal nitrides (TMNs) have attracted immense attention because of their high electronic conductivity, the unique electronic structure for appropriate adsorption and excellent catalytic ability [33, 34]. In recent years, 2D/2D heterostructures constituted via the face-to-face interaction of two substances are gaining popularity due to short electrons/ions diffusion distance and extra electrons transport pathways during reactions [35]. By reducing the thickness of bulk materials to ultrathin layers, the charge transport pathway will be minimized, thereby enabling high catalytic activity [36]. But until now, it is still a challenge to synthesize MXene-based 2D ultrathin heterostructured electrocatalysts, and the MXene-TMNs heterostructure has not reported so far.

Here, an in-situ and controllable nitridation method for synthesizing a hollow spherical structure consists of ultrathin 2D Ti_3_C_2_T_*x*_-TiN heterostructures (MX-TiN) was reported for the first time. The 2D heterostructures are close contacted between Ti_3_C_2_T_*x*_ MXene and TiN with a high-quantity interface region by the created bonds to motivate high-density electron flow, achieving an overall enhancement in electrical conductivity, which can provide low resistance, high electrochemical reaction kinetics. The TiN (001) surface shows superior electronic structure that exhibits favorable adsorptive ability and high catalytic activity. Moreover, the ultrathin layered heterostructure was controlled to several nanometers in thickness, which shortens the electrons diffusion distance from catalysts to reactive sulfur species and enlarges the surface area with abundant adsorptive and catalytic sites. According to DFT calculations, the LiPSs are strongly immobilized on the surface of MX-TiN by chemical bonds, and the reaction kinetics are greatly enhanced by reducing the conversion barrier from liquid Li_2_S_4_ to solid Li_2_S_2_/Li_2_S. From an overall perspective, the hollow spherical structure can accomplish high sulfur loading, suppress spontaneously restack of 2D structure, tightly trap the LiPSs inside the spheres, and accommodate large volume swelling during discharging. Benefiting from these advantages, the S/MX-TiN cathode displays remarkable initial capacity, high rate capability and outstanding long-term cyclability. More importantly, even with high sulfur loading and lean electrolytes, this advanced cathode achieves an impressive areal capacity with high Coulombic efficiency (CE).

## Experimental

### Synthesis of Ti_3_C_2_T_***x***_ MXene Suspension

Ti_3_C_2_T_x_ MXene nanosheets were prepared via an improved etching method. In detail, 1.0 g Ti_3_AlC_2_ MAX phase was added into a mixed solution of LiF/HCl and followed by stirring at 40 °C for 24 h. MXene suspension was obtained after water washing, ultrasonic processing, and centrifugation. It is worth noting that the MXene sheets here are micron-sized and required further ultrasonic treatment for 6 h to obtain the MXene nanosheets. Finally, the concentration of MXene nanosheets suspension was measured at ~ 5 mg mL^−1^. Pure MXene powder can be obtained by centrifugation after adding HCl and freeze-drying.

### Synthesis of Melamine–Formaldehyde Resin (MF) Spheres

Typically, 3.65 mL formaldehyde solution (37 wt% in water) was added into 80 mL deionized (DI) water and heated to 80 °C, then 1.0 g melamine was dissolved in the mixed solution. After stirring for 10 min, 0.1 mL formic acid was added to start polymerization and continued stirring at 80 °C for 1 h. Finally, the white precipitate was washed with DI water three times to obtain MF spheres.

### Synthesis of MX-TiN and MX-TiO_2_ and Fabrication of S-Containing Composites

Firstly, MF spheres were dispersed in DI water and mixed with MXene nanosheets. Due to electrostatic interaction, MXene is wrapped on MF spheres (MF@MXene). MF@MXene composites were transferred to an alumina ceramic crucible after freeze-drying. The composites were heated to 450 °C with a rate of 5 °C min^−1^ for 1 h to partially remove and carbonize the MF, followed by heating to 800 °C at 5 °C min^−1^ for 30 min under Ar/NH_3_ and finally MX-TiN was obtained after cooling. The MX-TiO_2_ was obtained in the same process under Ar atmosphere without extra NH_3_.

MX-TiN, MX-TiO_2,_ and pure MXene was separately mixed with sulfur powder at a mass ratio of 25:75. The mixture was transferred to a glass bottle and sealed with Ar gas. Then the bottles were heated to 155 °C for 15 h.

### Characterizations

The morphology of as-prepared samples was investigated by scanning electron microscopy (SEM, Hitachi S-4800) and transmission electron microscopy (TEM, JEM-2100F). X-ray diffraction (XRD) patterns were collected by a D/max-2550 PC XRD (Rigaku, CuKα radiation). X-ray photoelectron spectroscopy (XPS) spectra were obtained using Escalab 250Xi. The nitrogen adsorption/desorption isotherms were measured by ASAP 2020 instrument and the specific area was calculated by the BET method. Thermogravimetric analysis (TGA) was conducted in N_2_ using Discovery TGA Q5000IR from 50 to 500 °C at a heating rate of 5 °C min^−1^. The Raman spectra were measured by InVia Reflex (Renishaw) with a 532 nm laser.

### Visualized Adsorption Measurements

10 mM Li_2_S_4_ solution was prepared by dispersing Li_2_S and S (molar ratio of 1:3) powders in a mixture of DME and DOL (v/v = 1:1) under stirring at 60 °C overnight. Then 15 mg of MX-TiN, MX-TiO_2,_ and pure MXene powders were dispersed into 3 mL Li_2_S_4_ solution, respectively. After soaking for 8 h, the supernatants were collected for UV/Vis spectroscopy measurement (UV-1902PC, Phoenix). The sediments were washed with ethanol once and dried for XPS characterization.

### Assembly and Tests of Symmetric Cells

Active materials (MX-TiN, MX-TiO_2,_ and pure MXene) and PVDF with a mass ratio of 9:1 were dispersed in *N*-methylpyrrolidone (NMP) to form a uniform slurry, which was subsequently coated on carbon fiber paper (CP, 1 × 1 cm^2^) with a mass loading of 1.0 mg cm^−2^. The CP with active materials was used as both anode and cathode. Celgard-2400 membrane was employed as the separator. 50 μL of 0.1 M Li_2_S_4_ and 1 M LiTFSI in DOL/DME (v/v = 1:1) was used as electrolyte. The capacitance voltage (CV) tests of symmetric cells were performed within a potential range of − 1.0 to 1.0 V at a scanning rate of 3 mV s^−1^ on an electrochemical workstation (Metrohm Autolab). The electrolyte without Li_2_S_4_ was used as a blank control experiment.

### Li_2_S Precipitation and Decomposition Tests

Precipitation: The coin cells were assembled using the CP with active materials (MX-TiN, MX-TiO_2,_ and pure MXene; mass loading of 1 mg cm^−2^) as the cathodes, Li foil as the anodes, 30 μL 0.25 M Li_2_S_8_ in tetraglyme (cathode side) and 30 μL tetraglyme without Li_2_S_8_ (anode side) as electrolyte. The cells were first discharged galvanostatically at 0.112 mA to 2.06 V and then discharged potentiostatically at 2.05 V for 24,000 s for Li_2_S nucleation and growth.

Decomposition: The coin cells were assembled using the same method as in the Li_2_S precipitation tests. The cells were galvanostatically discharged to 1.8 V at a current of 0.1 mA, then galvanostatically discharged to 1.7 V at 0.01 mA to ensure the complete conversion of LiPSs into solid Li_2_S. Afterward, the cells were potentiostatically charged at 2.4 V until the current was below 0.01 mA to complete dissolve Li_2_S.

### Assembly and Electrochemical Tests of Li–S Cells

Active materials (S/MX-TiN, S/MX-TiO_2_, S/MXene), carbon black, and PVDF (mass ratio of 8:1:1) were mixed in NMP under stirring. The prepared uniform slurry was coated evenly on Al foil and followed by vacuum drying overnight. The average mass loading of sulfur is 1.0–1.2 mg cm^−2^ with excess electrolytes. In the high loading test, the mass loading of sulfur and the ratio of electrolyte-to-sulfur (E/S) were noted separately. Coin type cells (CR2032) were assembled using the Al foil with active materials as cathode, Li foil as the anode, Celgard-2400 as the separator, and 1.0 M LiTFSI in DOL/DME (*v*/*v* = 1:1) containing 2 wt% LiNO_3_ as electrolyte. Galvanostatic discharge–charge tests were carried out on a LANDHE Measurement System with a cut off voltage of 1.7–2.8 V. Current densities and specific capacities were calculated based on the mass of sulfur. CV measurements over the potential range of 1.7–2.8 V and EIS in the frequency of 0.01–100 kHz were conducted on an electrochemical workstation (Metrohm Autolab).

### Theoretical Calculation

The optimize geometries and electronic properties of all the investigated structures in this study were calculated at the density functional theory (DFT). Vienna ab initio simulation package (VASP) were employed in the simulations using the projector augmented wave (PAW) potentials with a plane wave cutoff of 400 eV. The Perdew Burke Ernzerhof (PBE) form of the exchange correlations functional was employed in the simulation. The heterostructure was constructed by combining the Ti_3_C_2_(OH)_2_ (001) surface with TiN (001) surface. Considering that our as-prepared sample is an approximately spherical structure, but the unit cell we simulated here is minuscule relative to the whole hollow sphere, so we ignore the effect of curvature and use the flat layered structure model. The slabs with 3 × 3 unit cells were modeled and a vacuum space exceeds 15 Å was employed to avoid the interaction between two periodic units. A 3 × 3 × 1 Monkhorst–Pack grid was used for sampling the Brillouin zones at all calculation. All the structures were optimized by using the conjugate gradient method, in which the convergence for total energy and interaction force was set to be 10^–5^ eV and 0.03 eV Å^−1^, respectively. The binding strength *E*_b_ of Li_2_S, Li_2_S_2_, Li_2_S_4_, Li_2_S_6_, and Li_2_S_8_ on the five investigated substrates were calculated as follows: *E*_b_ = (*E*_sub_ + *E*_ps_) − *E*_sub+ps_, where *E*_sub+ps_, *E*_ps_, and *E*_sub_ denote the calculated energies of the total adsorption system, adsorbates, and substrates, respectively.

## Results and Discussion

### Materials Synthesis and Characterization

**Fig. 1:**
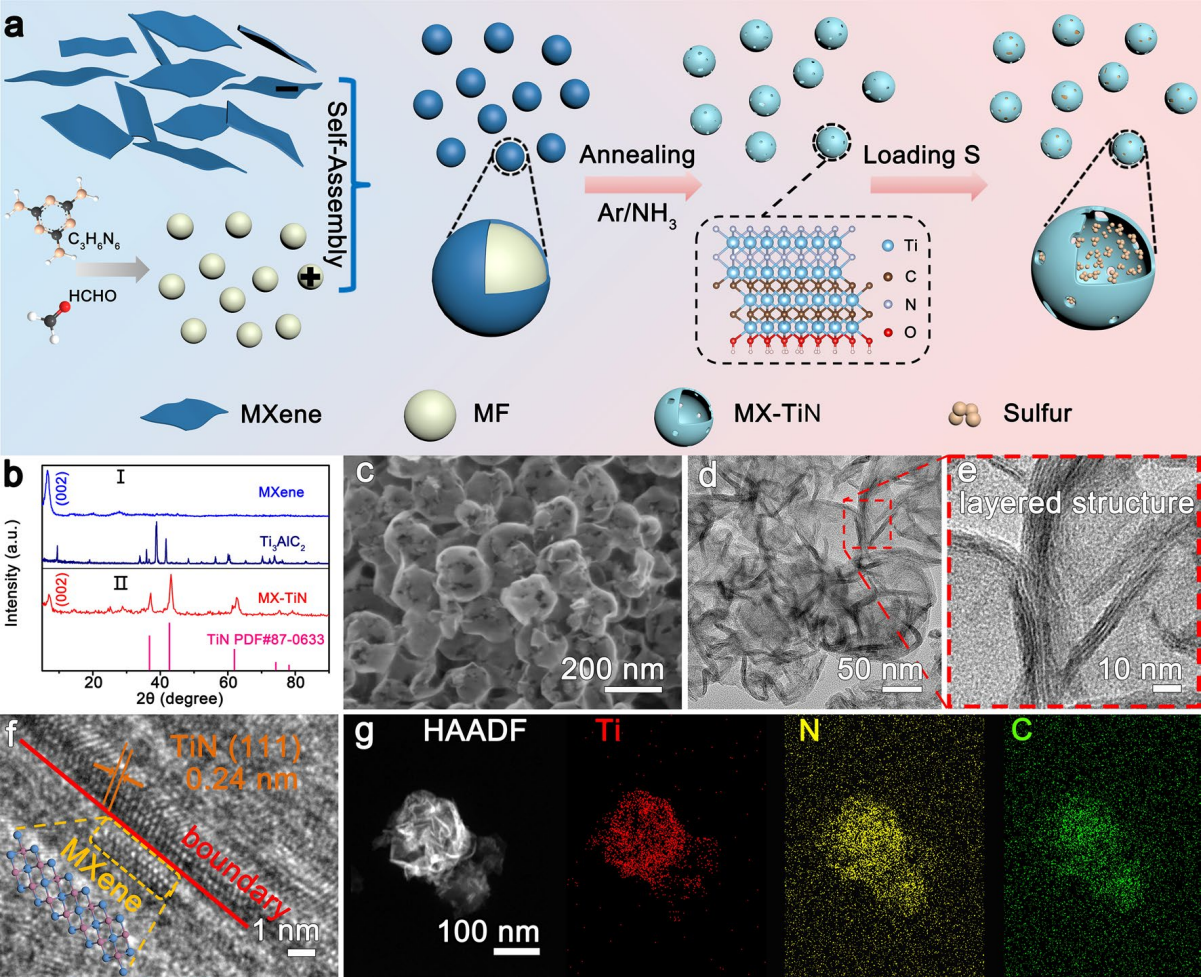
**a** Schematic illustration of synthetic procedures for MX-TiN and S/MX-TiN hybrid. **b** XRD patterns of Ti_3_AlC_2_, Ti_3_C_2_T_*x*_ MXene, and MX-TiN.**c** SEM and **d** TEM images of MX-TiN. **e** Enlarged TEM image of MX-TiN. **f** HRTEM image of Ti_3_C_2_T_*x*_-TiN heterostructure. **g** HAADF-STEM image and the corresponding elemental maps of MX-TiN

The synthesis process of hollow spherical structure composed of ultrathin 2D Ti_3_C_2_T_*x*_-TiN heterostructures and the S/MX-TiN composite is illustrated in Fig. 1a. As our previous work, the MAX phase Ti_3_AlC_2_ were etched by LiF/HCl mixture to selectively remove Al layers and followed by ultrasonication to produce Ti_3_C_2_T_*x*_ MXene nanosheets [37]. The multi-layer MXene has an accordion-like structure, while the few-layer MXene shows typical 2D ultrathin nanosheets by SEM and TEM (Fig. S1). MF spheres with a diameter of 200–300 nm are prepared by polymerization of melamine and formaldehyde triggered by formic acid (Fig. S2a). Since the surface of MXene is negatively charged due to its terminal groups, while the surface of MF is positively charged, there is a significant electrostatic interaction between MF spheres and MXene nanosheets (Fig. S3). MF spheres are uniformly wrapped by MXene spontaneously in mixed solutions with MXene suspension, forming MF@MX spheres (Fig. S2b). The lamellar structure of MXene with a few layers can be clearly observed at the edge of MF@MX, as shown in Fig. S2c–d. After the MF@MXene sphere was annealed in Ar/NH_3_ atmosphere at 800 °C for 30 min, the template was removed to form hollow spherical structures and the surface of MXene is successfully in-situ nitridation by controllable heat treatment process to form MX-TiN without destroying the typical 2D structure. Finally, sulfur was introduced into MX-TiN hollow space to produce S/MX-TiN hybrid by the melt-diffusion method.

As shown in Fig. 1b(I), the typical (002) peak at 7° in XRD patterns indicates the successful exfoliation of few-layer Ti_3_C_2_T_*x*_ MXene nanosheets. Moreover, the terminal groups of MXene are verified by Raman spectra, as shown in Fig. S4, the peaks at 203, 370, 582, and 620 cm^−1^ are related to Ti_3_C_2_ and its surface groups of A_1g_ Ti, C, O (Ti_3_C_2_O_2_), *E*_g_ O (Ti_3_C_2_O_2_), A_1g_ Ti, O (Ti_3_C_2_O_2_) and *E*_g_ C (Ti_3_C_2_(OH)_2_), respectively [1, 38]. After annealing in Ar/NH_3_ atmosphere at 800 °C for 30 min, the characteristic peaks of TiN (PDF#87-0633) appeared in XRD patterns (Fig. 1b(II)), meanwhile with the still presence of the (002) peak for MXene, which proves that the surface of MXene is partly in-situ nitrided to form TiN. The morphology and crystalline structure of MX-TiN were further observed by SEM and TEM. MX-TiN shows a nearly hollow spherical structure after removing the template (Figs. 1c and S5), which avoids the restacking of 2D nanosheets into densely packed structures or films. Besides, spherical structure of MX-TiN contributes to building more channels for fast electron/ion transport kinetics [39]. The TEM images at different magnifications in Figs. 1d and S6 also confirm the hollow spherical structure of MX-TiN and there is no obvious TiN particles. In a high magnified TEM image at the edge of MX-TiN (Figs. 1e and S6c), the typical layered structures with several nanometers in thickness can be observed, which retains the original ultrathin 2D geometry structure resembles MXene, demonstrating that the in-situ grown TiN arranged along the interior MXene to form ultrathin 2D heterostructures. As shown in the HRTEM image (Fig. 1f), the boundary of Ti_3_C_2_T_*x*_ layer and TiN layer can be observed at the atomic scale. At the lower left side of the boundary, the brighter Ti atoms form hexagonal structures, which belong to the typical structures of Ti_3_C_2_T_*x*_ MXene, while the interlayer spacing of 0.24 nm at the upper right corresponds to the TiN (111) crystal plane, which further proves the successful nitridation of the MXene to form ultrathin 2D Ti_3_C_2_T_*x*_-TiN heterostructures. The hollow spherical structure composed of ultrathin layers without aggregation ensures high sulfur loading, and large surface area providing plenty of adsorptive and catalytic active sites. However, the composition of MX-TiN is precisely controlled by the annealing time at Ar/NH_3_ (Fig. S7). When extended to 2 h, Ti_3_C_2_T_*x*_ MXene was fully transformed into TiN, which is confirmed by SEM images (Fig. S8) and the single phase of TiN without MXene (002) peak in XRD patterns (Fig. S9**)**. Based on these results, we conclude that NH_3_ molecules attack the surface of Ti_3_C_2_T_*x*_ MXene from outside to inside constantly under high temperature, and the layered TiN grow gradually to form the ultrathin 2D Ti_3_C_2_T_*x*_-TiN heterostructures. As seen from the high-angle annular dark-flied scanning transmission (HAADF-STEM) mappings (Fig. 1g), the Ti, N, and C elements are uniformly distributed throughout the sphere, revealing that the TiN is evenly decorated on the MXene without aggregation. After sulfur loading, the S/MX-TiN hybrid remains the spherical shape with similar sizes and morphologies. It should be noted that there is no obvious sulfur aggregation observed on the surface of S/MX-TiN hybrid from SEM images (Fig. S10), indicating that the sulfur was completely filled in the MX-TiN. Meanwhile, the XRD of the S/MX-TiN mainly shows the characteristic peaks of sulfur (PDF#96-2244) (Fig. S11a), and the elemental mapping of S/MX-TiN indicated the uniform distribution of elements Ti, C, N, and S (Fig. S12). For comparison, the MX-TiO_2_ with TiO_2_ aggregated into nanoparticles is synthesized by the same process under the Ar atmosphere without extra NH_3_ (Figs. S13-S15), and then the sulfur-containing composite S/MX-TiO_2_ is further prepared (Fig. S11b).

**Fig. 2:**
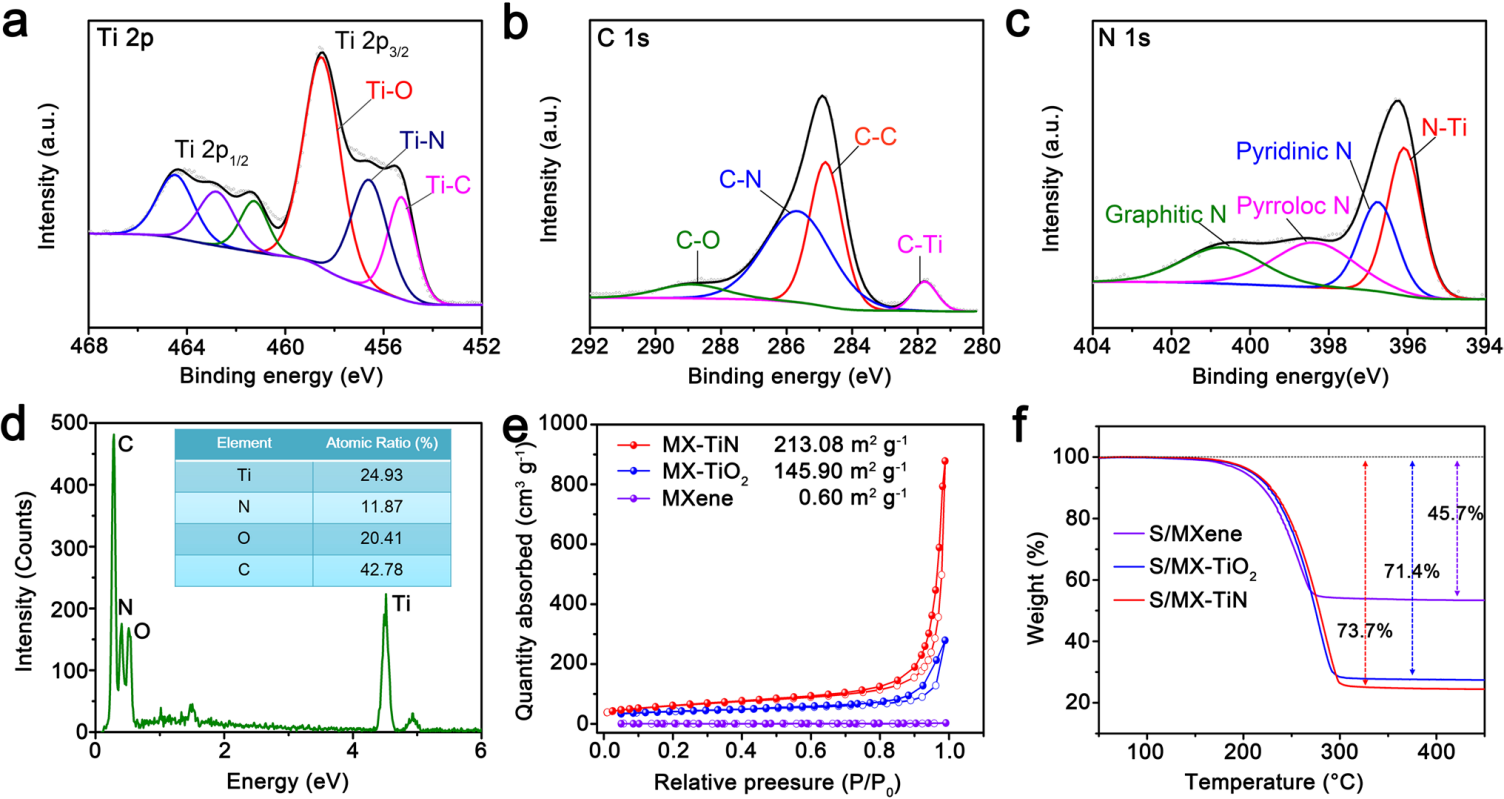
**a** Ti 2*p*, **b** C 1*s*, and**c** N 1*s* XPS profiles of MX-TiN. **d** Elemental compositions of MX-TiN by EDS analysis. **e** Nitrogen adsorption–desorption isotherms of MX-TiN, MX-TiO_2_, and MXene. **f** TGA curves of S/MXene, S/MX-TiO_2,_ and S/MX-TiN in Ar atmosphere

Figure 2a–c displays the XPS spectra of MX-TiN, which provide further information about the surface electron state and electronic structure. The high-resolution Ti 2*p* XPS spectra of MX-TiN show a pair of peaks (Ti 2*p*_3/2_ and Ti 2*p*_1/2_) (Fig. 2a), the signals at 455.3 and 461.3 eV can be ascribed to Ti-N configuration. The Ti-C peaks at 456.6 and 462.8 eV are derived from Ti_3_C_2_T_*x*_ MXene without nitridation, which is consistent with the obvious C-Ti peak (281.8 eV) of the high-resolution C 1*s* XPS spectra in Fig. 2b, proving the coexistence of Ti_3_C_2_T_*x*_ MXene, and TiN. Here, it should be noted that the Ti–O bonds in Ti 2*p* XPS are derived from the –O terminal groups of MXene and the inevitable surface oxidation [14, 40]. In addition, the N-Ti (396.1 eV) bond in N 1*s* spectra (Fig. 2c) also verifies the successful partial nitridation of MXene, another three peaks centered at 396.7, 398.4, and 400.7 eV can be assigned to pyridinic N, pyrrolic N, and graphitic N, respectively [41]. Raman spectroscopy in Fig. S16 also clearly shows the formation of MX-TiN with typical patterns of the two components of Ti_3_C_2_T_x_ MXene and TiN [33]. In addition, the XPS survey spectrum verifies (Fig. S17) the presence of Ti, C, and N elements, and the atomic ratio of Ti to N is 2.02. The ratio of Ti and N is 24.93 at% and 11.87 at% according to energy dispersive spectrometer (EDS) analysis (Fig. 2d), further indicating that the MXene is not fully nitridation, which is consistent with XPS analysis (Table S1).

In addition, the MX-TiN possesses a high specific surface area of 213.08 m^2^ g^−1^ based on BET method thanks to the hollow structure and the ultrathin 2D heterostructures, the value of which is much larger than those of reported MXene-based particle heterostructures (Fig. 2e and Table S2). In contrast, the surface area of MX-TiO_2_ and freeze-dried MXene nanosheets are smaller (145.90 and 0.6 m^2^ g^−1^, respectively) due to the aggregation of TiO_2_ nanoparticles and severe restacking of pure MXene nanosheets. It is well-known that both the adsorption and further conversion of sulfur species take place on the surface of catalysts [42], so the large surface area is vital to provide enough places for LiPSs adsorption and Li_2_S nucleation. In another concern, sulfur volatilization in the sulfur loading process by melting method causes the loss of active substances, so the sulfur content is one of the most important criteria to select sulfur host materials. The large specific surface area and hollow structure not only relieve the volumetric changes during cycling but also enable the high sulfur loading. As shown in Fig. 2f, the sulfur loading in the S/MX-TiN hybrid is 73.7 wt% (viz., only 2.3 wt% sulfur loss) determined by TGA, which is larger than those of S/MX-TiO_2_ (71.4%) and S/MXene (45.7%).

### Adsorptive and Catalytic Mechanism

**Fig. 3:**
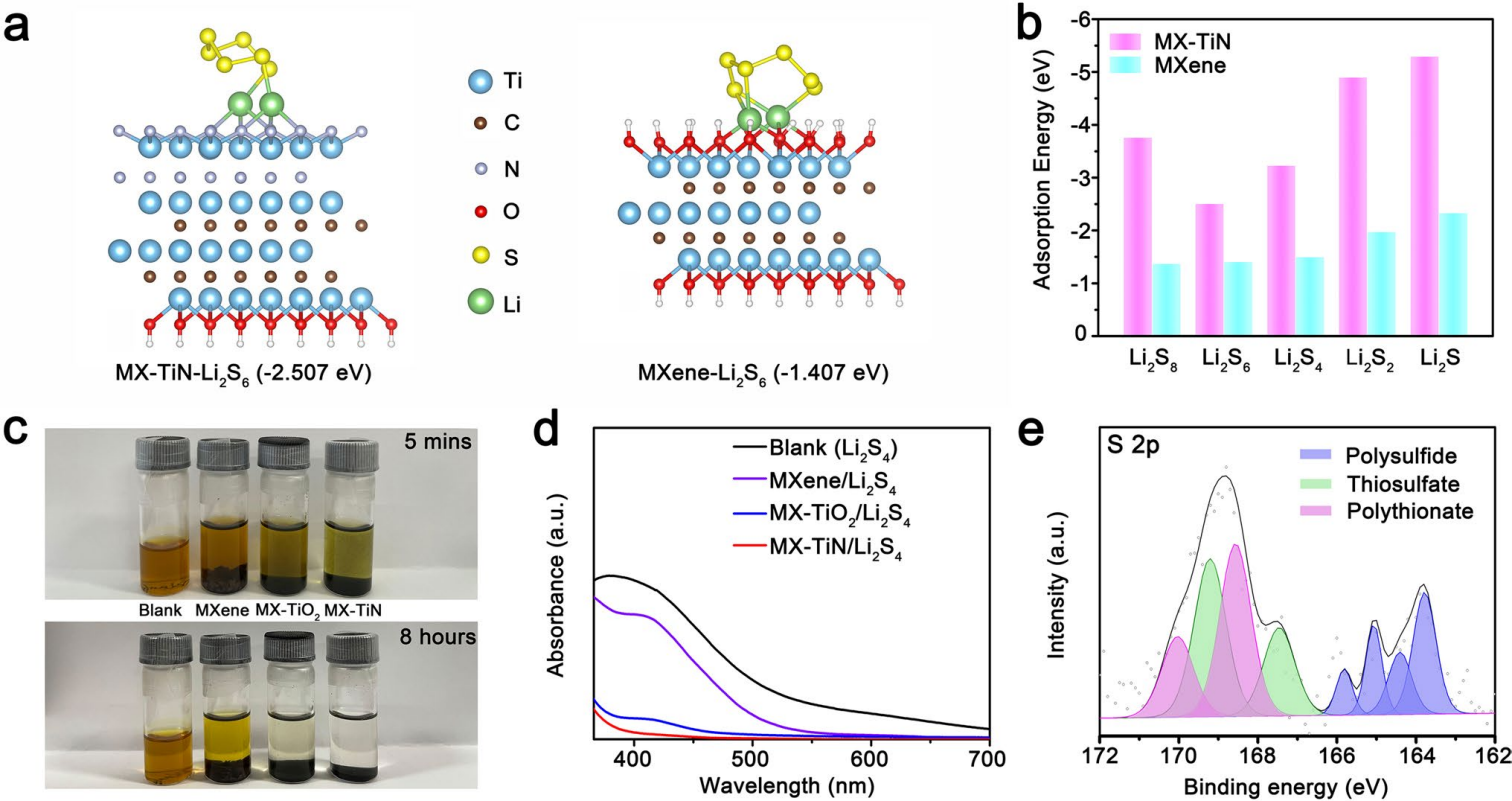
**a** Optimized configurations of Li_2_S_6_ on MX-TiN and Ti_3_C_2_T_*x*_ MXene. **b** Adsorption energies of different LiPSs species on MX-TiN and Ti_3_C_2_T_*x*_ MXene. **c** Optical photographs of pure Li_2_S_4_ solution, and MXene/Li_2_S_4_, MX-TiO_2_/Li_2_S_4,_ and MX-TiN/Li_2_S_4_ dispersions at 5 min and 8 h. **d** UV–Vis spectra of Li_2_S_4_ solutions after exposure at different adsorbents for 8 h. **e** S 2*p* XPS profile of MX-TiN/Li_2_S_4_ after soaking for 8 h

The strong adsorption ability of MX-TiN to LiPSs is the key to alleviating the shuttle effect. Firstly, the first-principle DFT calculations were performed to understand this chemisorption mechanism. The heterostructure was constructed by combining the TiN (001) surface on MXene surface. Figure 3a presents the side views of optimized configurations of Li_2_S_6_ adsorbed on MX-TiN and Ti_3_C_2_T_*x*_ MXene, and the configurations of other LiPSs species at different lithiation stages (Li_2_S_8_, Li_2_S_4_, Li_2_S_2_, and Li_2_S) are detailed in Fig. S18. In details, Li_2_S_6_ was immobilized by Li–N bonds on the MX-TiN surface with an adsorption energy of − 2.507 eV, while the adsorption energy on pure MXene is − 1.407 eV. As shown in Fig. 3b, it can be clearly observed that the adsorption energies of MX-TiN to all types of LiPSs are much higher than that of pure MXene, implying a stronger chemical interaction is formed between LiPSs and MX-TiN from a theoretical view.

To further verify the strong adsorption of MX-TiN towards LiPSs, the visual adsorption tests were performed by adding 15 mg sulfur host materials (MX-TiN, MX-TiO_2,_ and MXene) into 3 mL 10 mM Li_2_S_4_ solution. As shown in Fig. 3c, after soaking for 8 h, the solution containing MX-TiN becomes complete transparent, implying the strong adsorption ability. However, the solution containing of MXene is still slightly yellow after the same time. Ultraviolet–visible (UV–Vis) absorption spectrum was carried out to monitor the concentration differences of Li_2_S_4_ in the solution after 8 h (Fig. 3d) [43]. Notably, the order of remaining LiPSs intensity follows Blank > MXene > MX-TiO_2_ > MX-TiN, which trend is consistent with the visual adsorption tests. To further understand the reaction mechanism between MX-TiN and LiPSs, XPS was conducted after adsorption for 8 h. The high-resolution S 2*p* XPS spectra in Fig. 3e indicate the coexistence of polysulfide (LiPSs), thiosulfate ([S_2_O_3_]^2−^) and polythionate ([O_3_S_2_–(S)_x_–_2_–S_2_O_3_]). The formation of sulfur species besides polysulfide proves the strong interaction between LiPSs and MX-TiN, which is essential for realizing the fast-kinetic reaction of LiPSs conversion [34, 44].

**Fig. 4:**
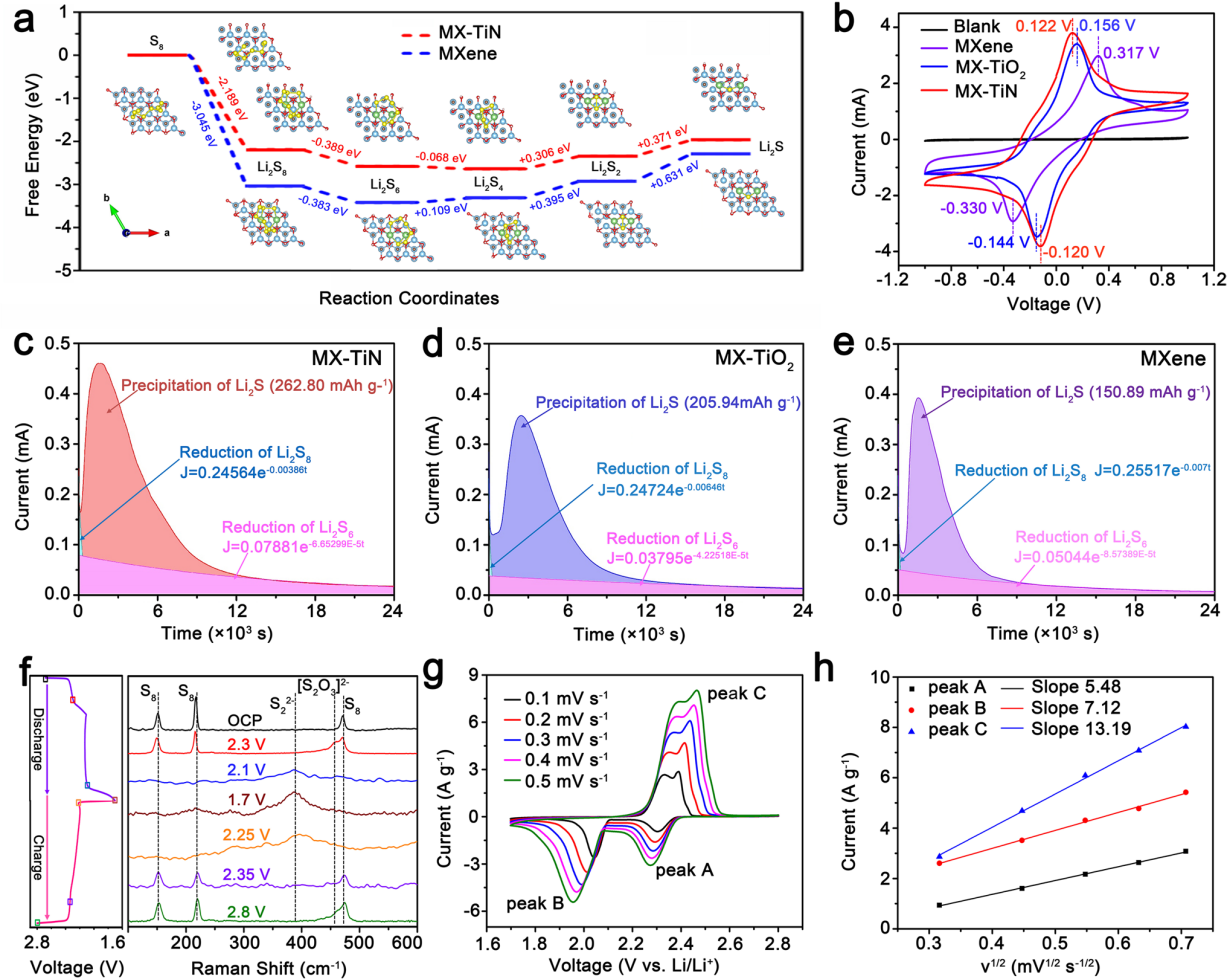
**a** The Gibbs free-energy profiles of LiPSs conversion on MX-TiN and pure MXene. **b** CV curves of MXene, MX-TiO_2,_ and MX-TiN symmetric cells with and without Li_2_S_4_ electrolyte. Potentiostatic discharge profile at 2.05 V on **c** MX-TiN electrode, **d** MX-TiO_2_ electrode, and **e** MXene electrode with Li_2_S_8_ electrolyte for evaluating the nucleation kinetics of Li_2_S. **f** First discharging/charging profiles and corresponding ex-situ Raman spectra at different potentials of S/MX-TiN cathode. **g** CV profiles at different scan rates of S/MX-TiN cathode and **h** corresponding *I*–*v*^2^ slope curves

The preferred sulfur host materials not only favor the adsorption of LiPSs, but also catalytically promote the redox reactions between LiPSs and Li_2_S_2_/Li_2_S, which can achieve efficient sulfur utilization and superior cycling performance for Li–S batteries. First, the electrocatalytic effect of MX-TiN to the conversion of LiPSs and nucleation of Li_2_S is theoretically confirmed by DFT calculations. The catalytic behavior is closely related to the intrinsic electronic structure properties of the heterostructures [45]. Figure 4a shows the Gibbs free-energy diagrams of the simplified reactions from S_8_ to Li_2_S on the MX-TiN and pure MXene. The reaction from S_8_ to Li_2_S_6_ is spontaneous and there is little difference for the conversion from Li_2_S_6_ to Li_2_S_4_. However, the reaction from Li_2_S_4_ to Li_2_S_2_/Li_2_S involves not only liquid–solid phase transitions but also slower solid–solid transitions, where requires an extremely high reaction free-energy, implying that this is the rete-controlling step and the origination of the sluggish sulfur redox reaction [46]. The reduction of Li_2_S_4_ and further of Li_2_S_2_ needs to obtain electrons, while the TiN (001) surface is dominated by metallic Ti-3d states, which is responsible for transmitting electrons from the high conductive MX-TiN matrix thus guaranteeing the efficient electrocatalytic activity. The reaction free-energy from Li_2_S_4_ to Li_2_S_2_ and finally to Li_2_S on the surface of MX-TiN is + 0.306 and + 0.371 eV, which is much lower than that of pure MXene (+ 0.395 and + 0.631 eV), indicating that MX-TiN effectively reduces the conversion barrier from Li_2_S_4_ to Li_2_S_2_/Li_2_S and thereby enhances the reaction kinetics. Note that the transition from Li_2_S_4_ to Li_2_S contributes to 75% theoretical capacity, suggesting that the effective catalytic ability is very important for the electrochemical performance in Li–S system.

To further demonstrate the catalytic effect of MX-TiN towards the conversion of LiPSs, symmetric cell tests and Li_2_S precipitation tests were carried out. Symmetric cells were assembled with 50 μL 0.1 M Li_2_S_4_ as the electrolyte to characterize the electrocatalytic effect of MX-TiN, MX-TiO_2_ and MXene for LiPSs conversion. As shown in Fig. 4b, the CV curves of MX-TiN exhibit a pair of redox peaks at − 0.120 and 0.122 V. These peaks can be attributed to the electrochemical conversion of Li_2_S_4_. The anodic peak is the reduction of LiPSs to Li_2_S_2_/Li_2_S. And in the cathodic scan, the Li_2_S_2_/Li_2_S was oxidized to LiPSs and finally to sulfur [47]. The MX-TiN symmetric cell without Li_2_S_4_ (Blank Group) exhibits a neat-zero capacitive current. In sharp contrast, the CV curves of MX-TiO_2_ and MXene electrodes display obviously lower current densities and more serious hysteresis voltage. These results strongly support the significant improvement of the electrocatalytic effect of MX-TiN to LiPSs conversion. Li_2_S precipitation tests are shown in Figs. 4c–e and S19, the profiles can be divided into contributions from the initial reduction of Li_2_S_8_, the bottom reduction of Li_2_S_6_, as well as the deposition of Li_2_S. According to Faraday’s law [7], MX-TiN electrode exhibits the highest precipitation capacity of 262.80 mAh g^−1^, implying higher catalytic efficiency for the precipitation of Li_2_S compared with those of MX-TiO_2_ (205.94 mAh g^−1^) and MXene (150.89 mAh g^−1^) electrodes. The catalytic ability of MX-TiN-2 h (single phase TiN) was also evaluated by Li_2_S precipitation test (Fig. S19), the current curve lies between that of MX-TiN and pure MXene, demonstrating the superiority of MX-TiN heterostructure. As we know, the precipitation of Li_2_S consists of crystal nucleation and crystal growth, and Li_2_S crystal nucleation in this process requires a higher driving force than its growth [48]. The peak current density and current response velocity can be used to evaluate the nucleation rate of Li_2_S. The ultrathin Ti_3_C_2_T_*x*_-TiN electrocatalysts shortens electrons diffusion distance, thus decreasing the transfer time from the MX-TiN to sulfur species. Therefore, MX-TiN electrode displays a higher current density (0.46 mA) and a faster current response at 1500 s than those of MX-TiO_2_ electrode (0.36 mA and 2460 s, respectively), indicating smaller nucleation barrier of Li_2_S on MX-TiN. The MXene electrode shows the lowest Li_2_S precipitation capacity, which can be blamed on weak adsorption ability and less nucleation sites due to its spontaneously restacking. To make things worse, the catalytic sites are inactivated as the insulating Li_2_S continuously covering on the surface of catalysts, and this problem is more serious under high sulfur loading. In order to prove that the ultrathin 2D MX-TiN can provide enough active sites for constant catalytic reactions, the cells were disassembled after 24,000 s potentiostatic discharge, the morphologies of deposited Li_2_S on the carbon fiber electrode were further observed by SEM (Fig. S20). The carbon fiber is uniform covered by a layer of Li_2_S on MX-TiN electrode, while the nonuniformly distributed Li_2_S particles can be observed on the other electrodes, which further proves the enhanced catalytic ability of MX-TiN for Li_2_S deposition. Meanwhile, the EDS mapping images also confirm the uniform Li_2_S coating on carbon fiber of the MX-TiN electrode (Fig. S19b). The decomposition of Li_2_S was further investigated by a potentiostatic charging process (Fig. S21), the MX-TiN electrode exhibits the highest capacity, demonstrating enhanced Li_2_S oxidation process. The above results clearly prove the bidirectional catalytic effect of MX-TiN for the conversion between LiPSs and Li_2_S.

To gain insight into the electrochemical reaction process during cycling, ex-situ Raman characterization of cycling electrodes was performed. In order to avoid oxidation, the electrodes were encapsulated in glass under Ar atmosphere before Raman test (Fig. S22). Figure 4f shows the first discharge–charge profiles and the corresponding ex-situ Raman spectra of S/MX-TiN cathode at some typical potentials. However, it should be noted that the peaks of MX-TiN are too weak to be witnessed compared to the strong peaks of sulfur species [49]. At the initial stage of discharge (OCP—2.30 V), the appeared weak peak of [S_2_O_3_]^2−^ close to the characteristic peaks of S_8_ demonstrates the gradual transformation of sulfur on MX-TiN [50], which is in line with the XPS results of Fig. 3e. As the discharge continued to 1.7 V, all of the other peaks disappeared and the strong peak of S^2−^ (Li_2_S) appeared gradually. It is proved that the efficient conversion from sulfur to Li_2_S under the catalytic effect of MX-TiN, which improves the utilization of active materials. The opposite process can be observed in the charging stage, implying the high reversibility of conversion reaction.

In order to demonstrate the enhanced reaction kinetics by MX-TiN, we studied the diffusion coefficient of lithium-ion. As shown in Figs. 4g and S23a–c, CV curves of different cathodes at incremental scanning rates are performed. In the cathodic scan process, there are two reduction peaks which can be ascribed to the reduction of S_8_ to soluble LiPSs (peak A), and subsequent reduction of LiPSs to solid Li_2_S_2_/Li_2_S (peak B). In the reversible anodic scan, Li_2_S_2_/Li_2_S are oxidized to LiPSs, and finally to S_8_ (peak C). Notably, the cathode with MX-TiN displayed a higher current density and a lower reaction polarization than those of MX-TiO_2_ and MXene. In addition, as the scan rate increases, MX-TiN cathode maintains clear CV curves and sharp redox peaks, indicating enhanced reaction kinetics and good rate performance. However, as for S/MX-TiO_2_ and S/MXene cathodes, the current increase slowly and the redox peaks become blurred as the scan rate increases. The diffusion coefficient of lithium-ion (*D*_*Li*_^+^) can be calculated by the Randles–Sevcik equation, *I* = 2.686 × 10^5^*n*^1.5^AD_Li_^+^Cv^0.5^, where *I* is the peak current, *n* is the number of charges transferred, *A* is the geometric area of electrode, *D*_Li_^+^ is diffusion coefficient of lithium-ion, *C* is the concentration of lithium-ion in electrolyte, and *v* is the scanning rate. The peak current *I* has a good positive correlation with the square root of scan rate *v* (Figs. 4g and S23b, d), and the slopes of *I–v*^0.5^ for S/MX-TiN cathode are much bigger than those of S/MX-TiO_2_ and S/MXene, demonstrating the faster lithium-ion diffusion. The rapid lithium-ion diffusion can promote sulfur utilization and improve high-rate performance. Furthermore, the *D*_*Li*_^+^ of S/MX-TiN cathode at redox peaks A, B, C are 1.75 × 10^–7^, 2.96 × 10^–7^, 10.14 × 10^–7^ cm^2^ s^−1^, respectively, well above the *D*_*Li*_^+^ values for S/MX-TiO_2_ and S/MXene, further indicating the fast reaction kinetics (Fig. S24). We concluded that the larger *D*_Li_^+^ measured in S/MX-TiN cathode was due to the excellent conductive skeletons constructed by MX-TiN, which provides the fast channels for lithium-ions transport.

**Fig. 5:**
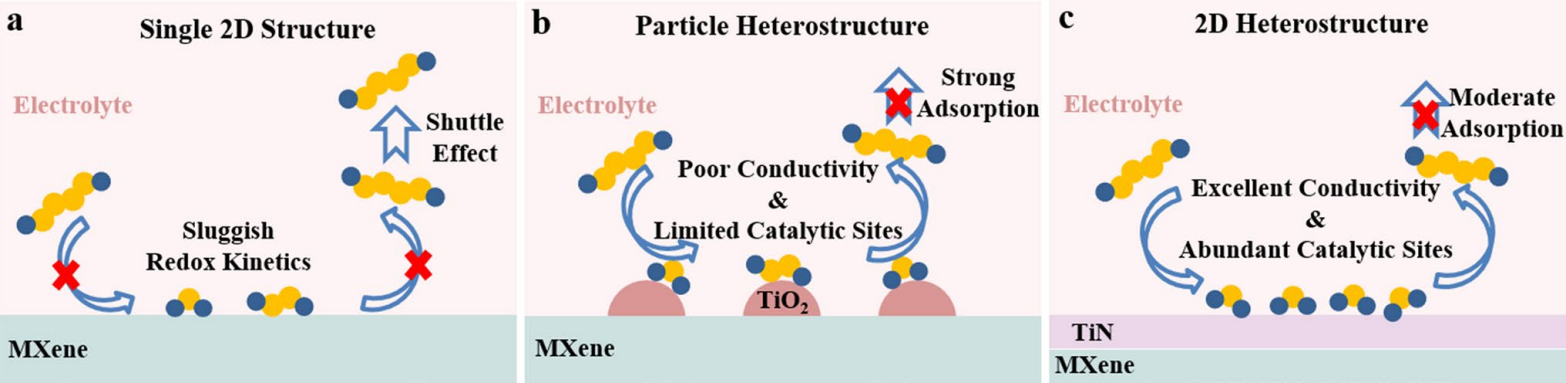
Schematic illustration of adsorptive and catalytic mechanism of LiPSs species by different structures of **a** pure MXene, **b** MX-TiO_2_, and **c** MX-TiN

Taken all theoretical calculations and experimental results together, Fig. 5 schematically illustrates the adsorptive and catalytic mechanism of different sulfur species on (a) pure MXene, (b) MX-TiO_2_, and (c) MX-TiN. Firstly, the hollow spherical structure of MX-TiN derived from template-removal method prevents the restacking of 2D heterostructures, enabling sufficient space to store sulfur and accommodate the large sulfur expansion. Secondly, the construction of ultrathin thin layered structure not only exhibits the large surface area but also constructs fast channels for electrons and ions transport. In principle, a larger surface area will provide more interface contacting sulfur species. Therefore, compared with MX-TiO_2_ and pure MXene, MX-TiN with ultrathin layered 2D structures possess the most adsorptive and catalytic sites and the shortest electron transfer distances from catalysts to sulfur species. Thirdly, the metallic TiN (001) surface makes the heterostructures exhibit better conductivity and superior electronic structure, which reduces the barriers of LiPSs conversion and Li_2_S nucleation and enhances the catalytic activity, thus greatly boosting the Li–S reaction kinetics. And MX-TiN shows greater interfacial interaction to LiPSs, which captures the LiPSs strongly and effectively mitigates the serious shuttle effect.

### Electrochemical Performance

**Fig. 6:**
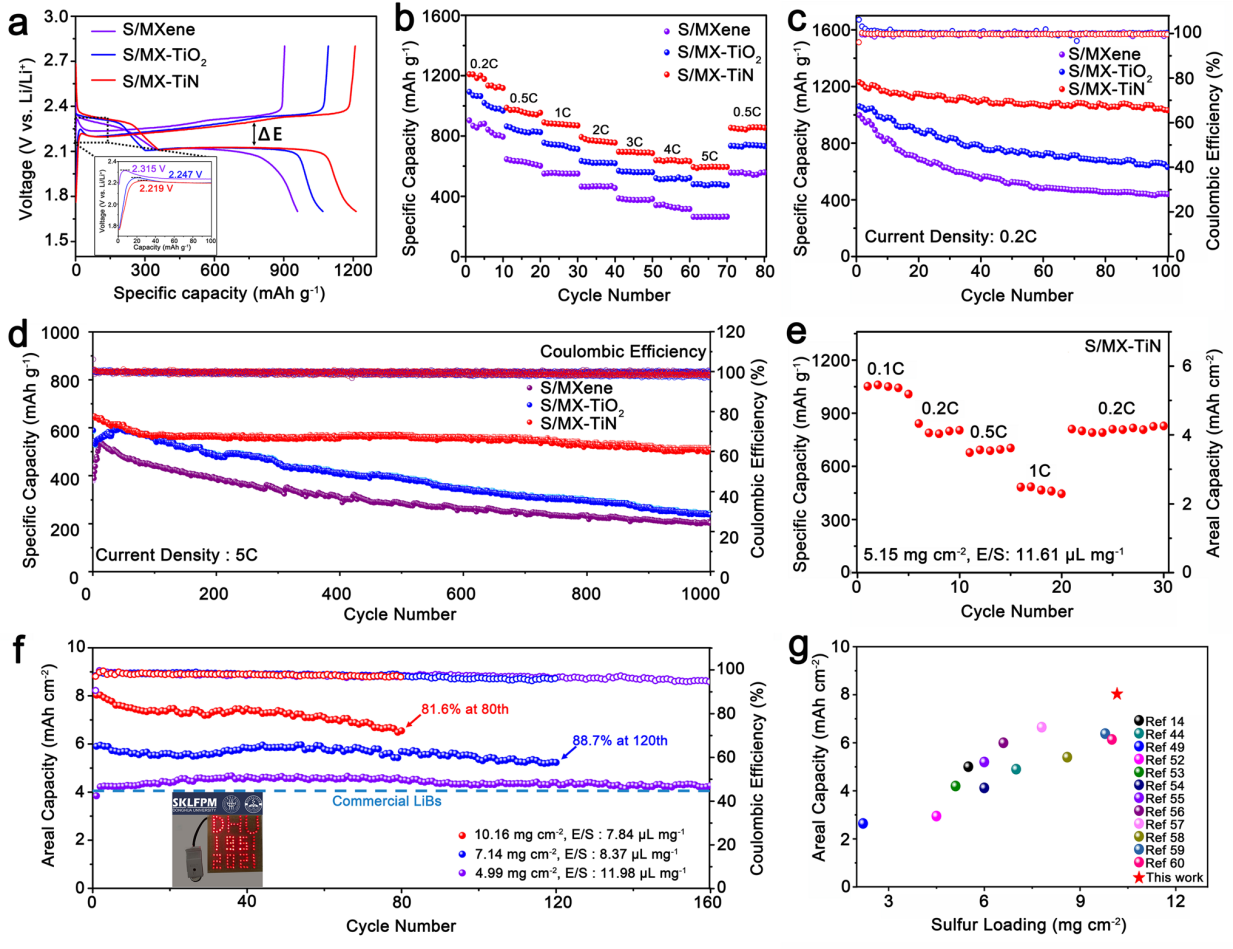
**a** The initial discharge–charge profiles of S/MXene, S/MX-TiO_2,_ and S/MX-TiN cathodes. **b** Rate performance and **c** cycling performance at 0.2C of S/MXene, S/MX-TiO_2,_ and S/MX-TiN cathodes. **d** Long-term cyclability of S/MX-TiN, S/MX-TiO_2_, and S/MXene at 5C. **e** Rate capability of S/MX-TiN at a high sulfur loading of 5.15 mg cm^−2^ and E/S ratio of 11.61. **f** Cycling performance of S/MX-TiN cathode at different sulfur loading of 4.99, 7.14, and 10.16 mg cm^−2^, respectively. Inset: Optical photograph of a “DHU 1951 2021” shape composed of 115 LED lamps. **g** Comparison of sulfur loading and areal capacity of S/MX-TiN cathode with other reported MXene-based and titanium-based sulfur cathodes

Coin cells were assembled with lithium metal as anode and S/MX-TiN, S/MX-TiO_2_, S/MXene as cathodes to evaluate their electrochemical performance. Figure 6a displays the first discharge–charge profiles at a current density of 0. C. The initial capacity of S/MX-TiN is 1229.9 mAh g^−1^, much higher than those of S/MX-TiO_2_ (1060.2 mAh g^−1^) and S/MXene (997.0 mAh g^−1^). It is worth noting that Li_2_S decomposition is the first step for the charging process, and the conversion of Li_2_S to LiPSs leads to phase transformations and requires a large overpotential as the driving force to overcome the Li_2_S dissociation barrier. The S/MX-TiN cathode exhibits the smallest polarization (Δ*E*) and the lowest overpotential for Li_2_S oxidation (inset of Fig. 6a), which can strongly support efficient oxidation of Li_2_S and high cyclic reversibility. In contrast, the larger ΔE and overpotential of S/MX-TiO_2_ and S/MXene lead to accumulation of Li_2_S, even worse the nondecomposable Li_2_S will passivate the electrode and decrease the utilization of sulfur [51]. Meanwhile, Fig. S25 shows similar galvanostatic discharge–charge profiles of different cathodes at various current densities from 0.2 to 5C. The S/MX-TiN delivered a stable plateau at elevated current densities due to enhanced reaction kinetics, thus leading to superior rate performance and high CE. In sharp contrast, the S/MX-TiO_2_ and S/MXene electrodes present much larger polarization and the vanishing plateau under high C-rates. The rate performance was measured by increasing current density every ten cycles (Fig. 6b). The S/MX-TiN cathode exhibits high capacities of 1161.1, 960.4, 878.1, 768.1, 691.7, 635.9, 593.9 mAh g^−1^ (average capacities) at current densities from 0.2C, 0.5C, 1C, 2C, 3C, 4C to 5C, respectively. In comparison, the S/MX-TiO_2_ and S/MXene electrode deliver only 477.3 and 263.8 mAh g^−1^ at the high current rate of 5 C.

Galvanostatic cycling stability performance was evaluated at different current densities. As shown in Fig. 6c, the S/MX-TiN delivers a reversible capacity of 1028.2 mAh g^−1^ with a good capacity retention of 87.1% after 100 cycles at 0.2C, which is much higher than those of S/MX-TiO_2_ (632.9 mAh g^−1^, 65.6%) and S/MXene (443.9 mAh g^−1^, 45.9%). The voltage plateaus of S/MX-TiN cathode change slightly after 100 cycles (Fig. S26a), indicating the high-capacity reversibility. However, there is a sharp capacity decrease with the increase of polarization for S/MX-TiO_2_ and S/MXene cathode (Fig. S26b-c). To further explore the long cyclic stability at a high current density, S/MX-TiN was tested at 5C over 1000 cycles (Fig. 6d). The S/MX-TiN displays an initial specific capacity of 647.7 mAh g^−1^, and maintains a capacity of 516.9 mAh g^−1^ after 1000 cycles with the CE as high as 99.3%, corresponding to 79.8% capacity retention and an average capacity decay of as low as 0.022% per cycle, which is much better than that of S/MX-TiO_2_ and S/MXene. The capacity of pure MX-TiN was tested under the same conditions to avoid its interference, its capacity contribution to the total capacity is negligible (Fig. S27). Furthermore, according to the CV measurements (Fig. S28), MX-TiN remains electrochemical inert over a voltage range of 1.7–2.8 V.

Electrochemical impedance spectra (EIS) of different cathodes were carried out on fresh batteries. As shown in Fig. S29a, the EIS consists of a semicircle at high frequency region and an inclined line on low frequency region. The intersection between the initial part of the semicircle and the horizontal axis represents the electrolyte resistance (*R*_e_). And the diameter of the semicircle represents the charge transfer resistance (*R*_ct_), which reflects the reaction kinetics of electrolyte and electrode interface. The equivalent circuit was performed (Fig. S29b) and the simulated results are summarized in Table S3. The S/MX-TiN exhibits the lowest *R*_ct_ compared to the other two cathodes, demonstrating the smaller charge-transfer barrier at the MX-TiN heterostructured interface and enhanced interfacial redox kinetics. Moreover, to explore the changes of lithium metal anode and cathode of different cells during cycling, we disassembled the cells after cycling and characterized the surface morphology of anodes and cathodes by SEM (Figs. S30–S31). Thanks to the good adsorption and efficient catalytic effect of MX-TiN towards LiPSs, there is only a small amount of speckled Li_2_S deposited on the surface of lithium metal after cycling. Meanwhile, the surface of S/MX-TiN cathodes remains flat after cycling due to the buffering ability of the hollow structure, while the S/MXene cathode is cracked. Therefore, we conclude that the MX-TiN alleviates the shuttle effect and catalyzes the rapid conversion of LiPSs, which accounts for the remarkable cycling performance for S/MX-TiN cathode. In sharp contrast, lots of irregular sediments appeared on the surface of lithium for the cell with S/MXene cathode after cycling, which is caused by the LiPSs shuttle through the separator and form an insulating Li_2_S layer on the surface of lithium metal directly. This results in a rapid decline in capacity and an increase in resistance.

To broaden the practical applications of S/MX-TiN electrode, the evaluations of high-sulfur loading with lean electrolytes should be critically considered. We first investigate the wettability of electrolyte with S/MX-TiN cathode by testing contact angle. As shown in Fig. S32, the contact angle becomes less than 5° immediately when the electrolyte contacts the electrode, and the electrolyte completely infiltrate the electrode within a few seconds, implying the excellent wettability of electrolyte and S/MX-TiN cathode. Figure 6e shows the rate performance of S/MX-TiN cathode at a high-sulfur loading of 5.15 mg cm^−2^ and an* E/S* ratio of 11.61 μL mg^−1^. The S/MX-TiN electrode exhibits excellent rate capacities of 1042.3, 802.9, 690.8, 467.6 mAh g^−1^, the corresponding areal capacities of 5.36, 4.14, 3.56, 2.41 mAh cm^−2^ at 0.1C, 0.2C, 0.5C, and 1C, respectively. Notably, as the discharge rate decreases from 1C to 0.2C, the capacity returns to 808.1 mAh g^−1^ (4.16 mAh cm^−2^), conferring good reversibility at a relatively high-sulfur loading. Accordingly, the discharge–charge profiles of S/MX-TiN have a stable plateau at different current densities (Fig. S33), indicating its excellent reaction kinetics even at a high-sulfur loading. Moreover, Fig. 6f displays the cycling performance of S/MX-TiN cathodes at the different sulfur loading and E/S ratio at 0.2C. Impressively, the cathode with lean electrolytes (*E*/*S* = 7.84) and 10.16 mg cm^−2^ sulfur loading exhibits an initial capacity of 8.27 mAh cm^−2^ and 81.6% capacity retention with an average CE of 97.7% after 80 stable cycles. The cathode with 4.99 mg cm^−2^ and a slightly higher electrolyte (*E*/*S* = 11.98) exceeds 4 mAh cm^−2^ (capacity of typical commercial lithium-ion batteries) even after 160 cycles. Furthermore, a fresh cell battery with S/MX-TiN cathode (sulfur loading: ~ 5 mg cm^−2^) can light up 115 light-emitting diode (LED) lamps with the words “DHU 1951 2021” (inset Fig. 6f). Evidently, the obtained electrochemical performance in terms of sulfur loading and areal capacity show considerable competitiveness compared with other MXene-based and titanium-based sulfur host materials (Fig. 6g) [14, 44, 49,52–60].

## Conclusions

In summary, we rationally designed and synthesized a novel electrocatalyst for high-performance Li–S batteries based on ultrathin 2D Ti_3_C_2_T_*x*_-TiN heterostructure. We explained the enhanced catalytic performance from the aspects of interfacial areas, sulfur species binding energy, polysulfides conversion and Li_2_S nucleation barriers, and lithium-ion diffusion coefficient. By constructing the ultrathin layered heterostructures within the thickness of several nanometers, MX-TiN exposes large surface area up to 213.08 m^2^ g^−1^ with abundant adsorptive and catalytic sites, shortens the electrons transfer distances to the reactive sulfur species. The metallic TiN (001) surface exhibits excellent conductivity and superior electronic structures, favoring a greater interaction with polysulfides and reducing the conversion barriers between LiPSs and Li_2_S. Meanwhile, the internal hollow structure of MX-TiN provides enough room for high sulfur loading and accommodates the large volume expansion of active materials during discharging, guaranteeing the good electrical contact and stability of the electrodes. On the basis of these fundamental understandings, the S/MX-TiN cathode exhibits low polarization, high initial capacity, and long-term cycling stability with extremely low-capacity decay rate. More importantly, the cathode delivers impressive area capacity (8.27 mAh cm^−2^) under high sulfur loading (10.16 mg cm^−2^) and lean electrolytes (*E*/*S* = 7.84), which improves the feasibility for practical applications of Li–S batteries. With insights generated by this work, we expect the rational design and construct of ultrathin 2D heterostructures not only improve the performance of Li–S battery system, but also broaden the horizons of electrocatalyst design for other energy storage and conversion systems.

## Supplementary Information

Below is the link to the electronic supplementary material.

Supplementary file1 (PDF 2841 kb)
