# Supplementary material for: Construction of Ultrathin Layered MXene-TiN Heterostructure Enabling Favorable Catalytic Ability for High-Areal-Capacity Lithium–Sulfur Batteries
Source: Nanomicro Lett. 2022 Sep 17;14:189. doi: 10.1007/s40820-022-00935-0 (PMC9482562; doi:10.1007/s40820-022-00935-0)
Supplement: Supplementary file 1 — Supplementary file1 (PDF 2841 kb) [file 40820_2022_935_MOESM1_ESM.pdf]

Supporting Information for

Construction of Ultrathin Layered MXene-TiN Heterostructure Enabling Favorable Catalytic Ability for High-Areal-Capacity Lithium-Sulfur Batteries

Hao Wang[1], Zhe Cui[1], Shu-Ang He[1], Jinqi Zhu[1], Wei Luo[1,]*, Qian Liu[2] and Rujia Zou[1,]*

[1] State Key Laboratory for Modification of Chemical Fibers and Polymer Materials, College of Materials Science and Engineering, Donghua University, Shanghai 201620, P. R. China

[2] Department of Physics, College of Science, Donghua University, Shanghai 201620, P. R. China

*Corresponding authors. E-mail: rjzou@dhu.edu.cn (Rujia Zou), wluo@dhu.edu.cn (Wei Luo)

Supplementary Figures and Tables

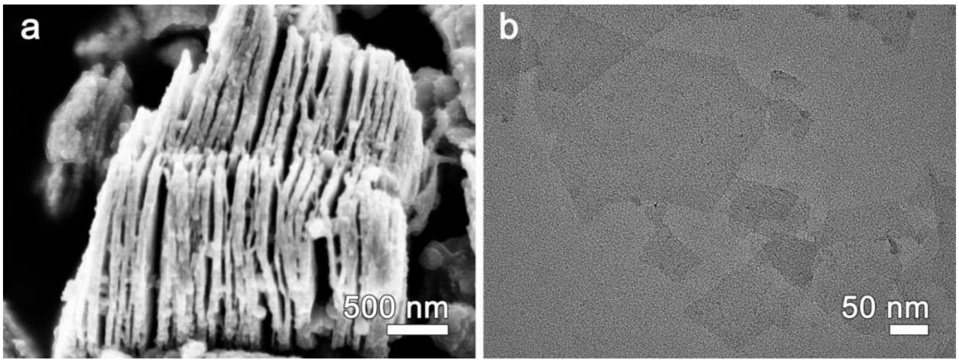

Fig. S1 (**a**) SEM image of multi-layer MXene. (**b**) TEM image of few-layer MXene

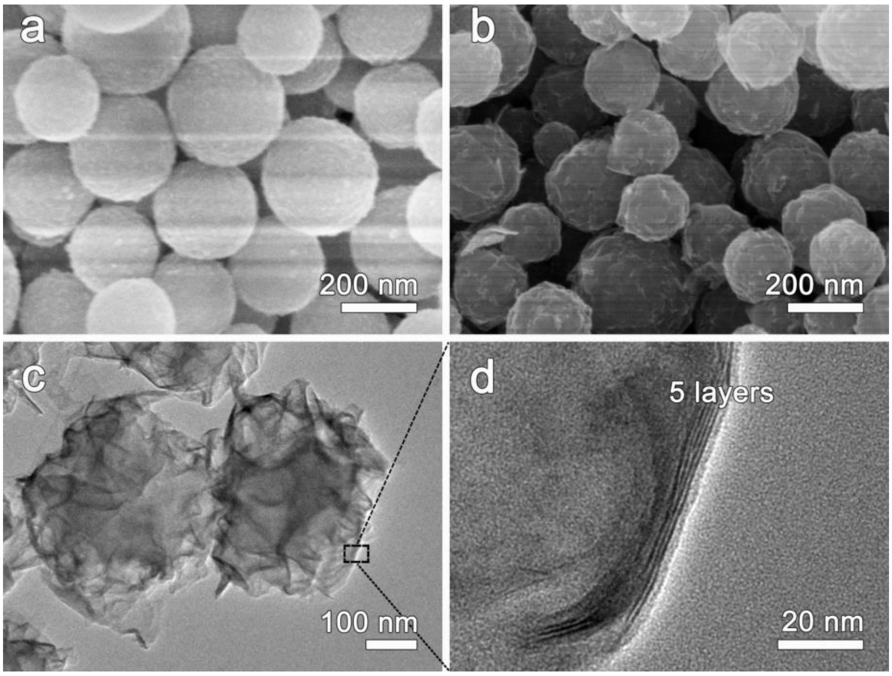

Fig. S2 (**a**) SEM images of MF spheres, (**b**) SEM, (**c**) TEM and (**d**) HRTEM images of MF@MX

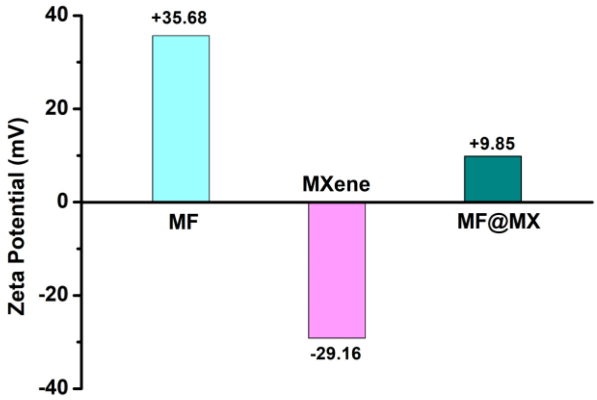

Fig. S3 The mean zeta potential of MF, MXene and MF@MX

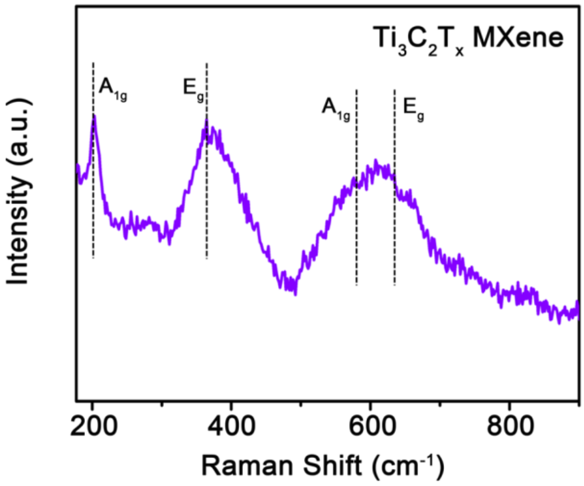

Fig. S4 Raman spectrum of $Ti_3C_2T_x$ MXene

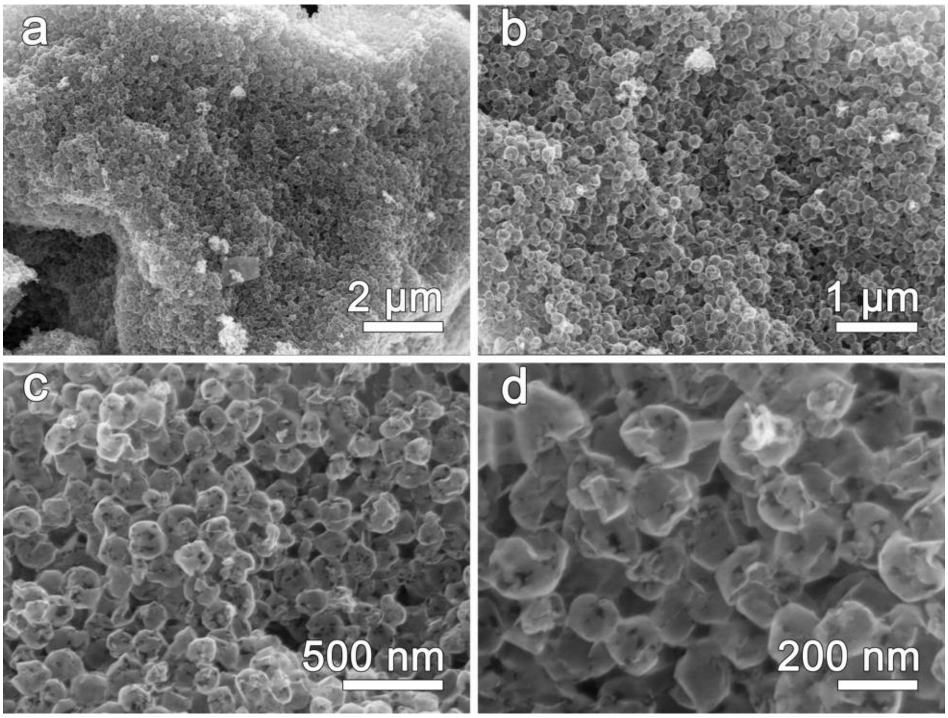

Fig. S5 (**a-d**) SEM images of MX-TiN spheres at different magnifications

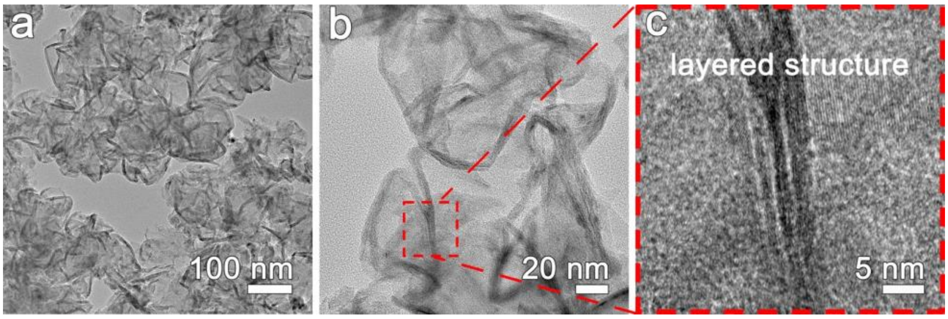

Fig. S6 (**a, b**) TEM images of MX-TiN at different magnifications. (**c**) HRTEM image of the layered structure at the edge of MX-TiN sphere

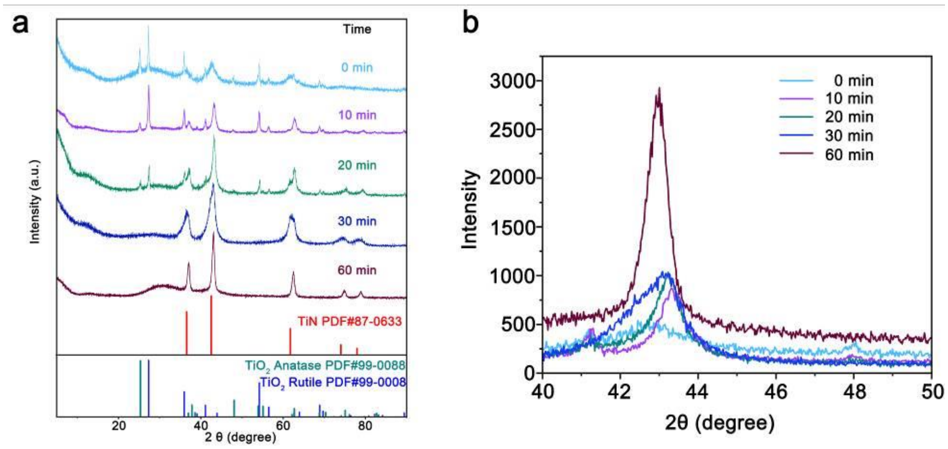

Fig. S7 (**a**) XRD patterns of samples annealing under NH_3 for different time. (**b**) The comparison of TiN (200) peak during annealing process

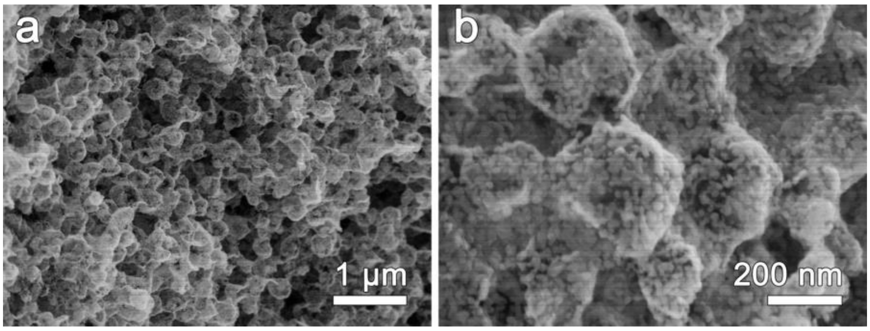

Fig. S8 (**a, b**) SEM images of MX-TiN-2h (2 hours' annealing at Ar/NH_3) at different magnifications

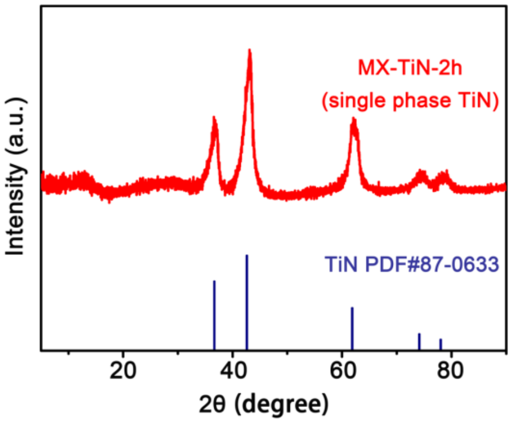

Fig. S9 After annealing at Ar/NH_3 for 2 h, the XRD patterns show the single phase of TiN

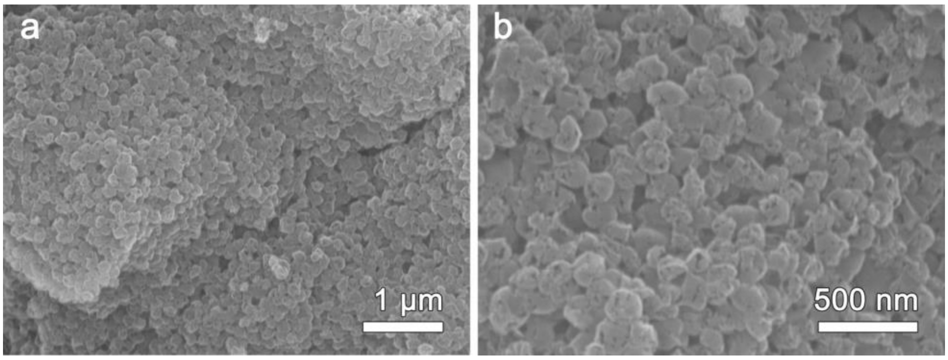

Fig. S10 (**a, b**) SEM images of S/MX-TiN different magnifications

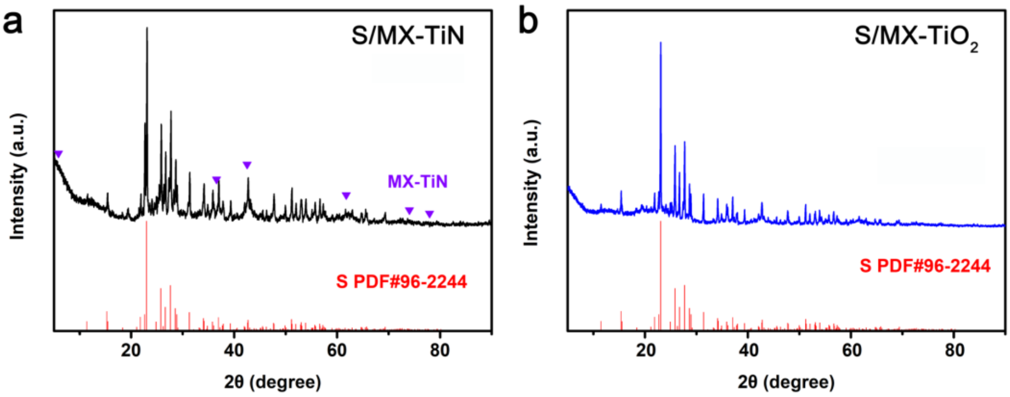

Fig. S11 XRD patterns of (**a**) S/MX-TiN composite and (**b**) S/MX-TiO_2 composite

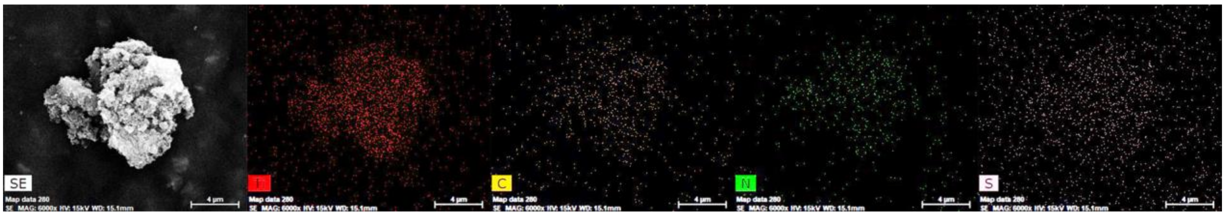

Fig. S12 SEM image of S/MX-TiN composite and the corresponding elemental mapping of Ti, C, N, and S elements

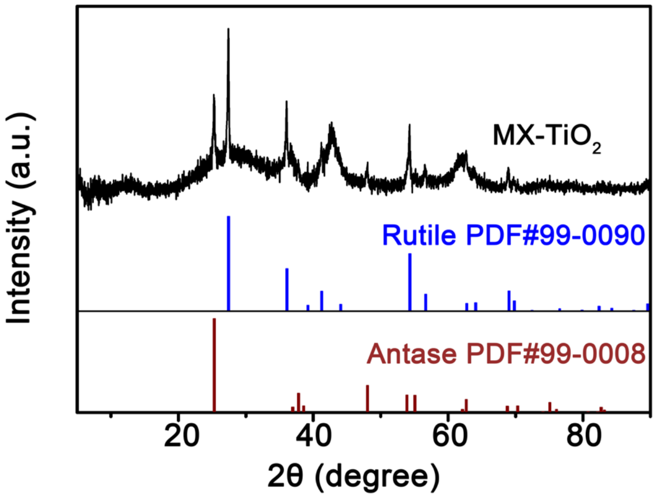

Fig. S13 The XRD patterns of MX-TiO_2 correspond to the anatase phase (PDF#99-0008) and rutile phase (PDF#99-0090), respectively

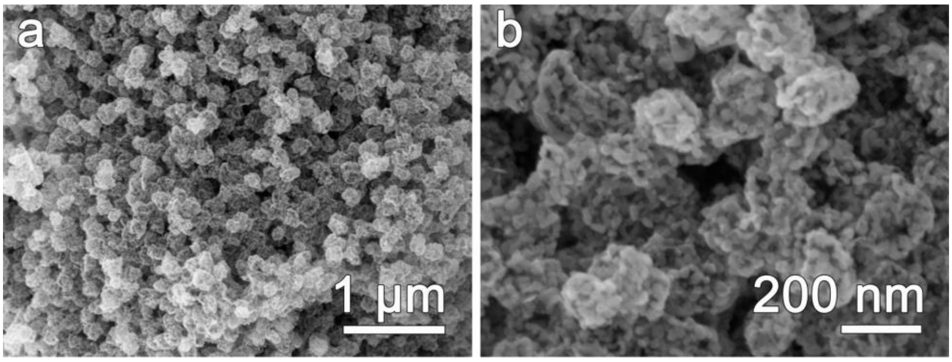

Fig. S14 (**a, b**) SEM images of MX-TiO_2 at different magnifications. TiO_2 nanoparticles with a diameter of 20-40 nm can be clearly observed

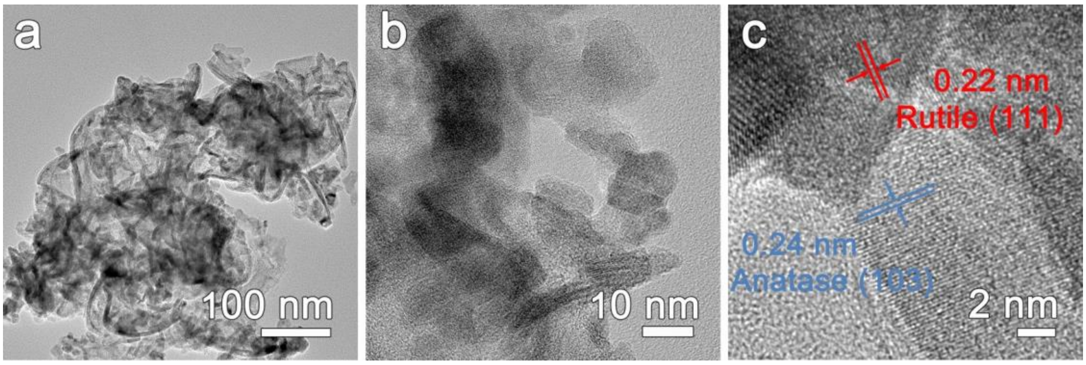

Fig. S15 (**a, b**) TEM images of MX-TiO_2 at different magnifications. (**c**) HRTEM image of MX-TiO_2. HRTEM also confirmed that the crystal phase of MX-TiO_2 is a mixture of anatase and rutile, which corresponds to the XRD

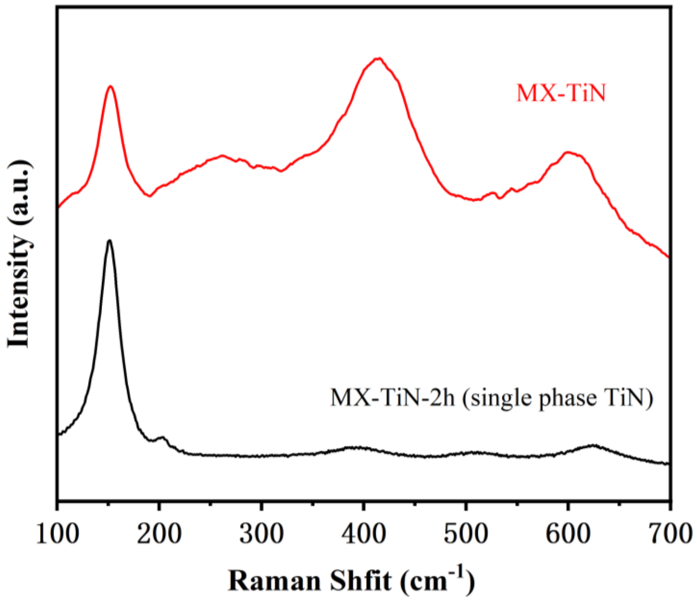

Fig. S16 Raman spectroscopy for the as-synthesized of MX-TiN and MX-TiN-2h (single phase TiN)

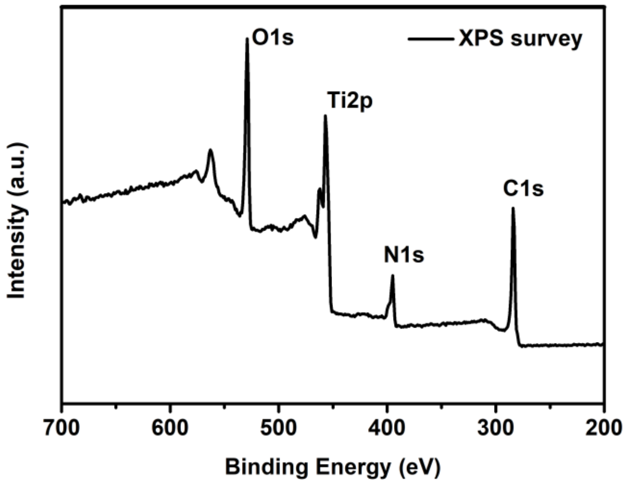

Fig. S17 XPS survey spectrum of MX-TiN

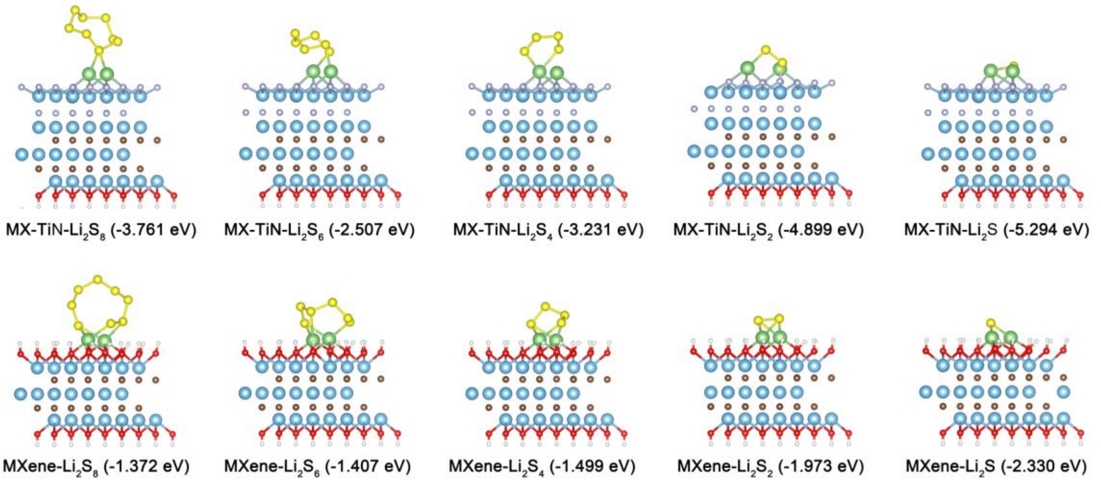

Fig. S18 Optimized configurations of different LiPS species (Li_2S_8, Li_2S_6, Li_2S_4, Li_2S_2, and Li_2S) on MX-TiN and pure $Ti_3C_2T_x$ MXene

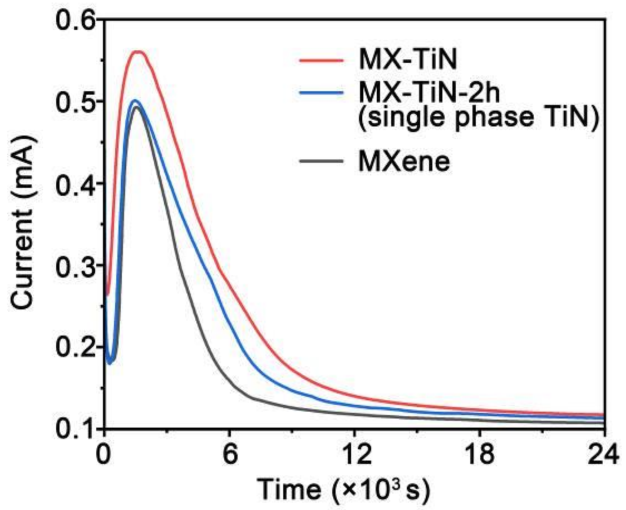

Fig. S19 The Li_2S precipitation test of MX-TiN, MX-TiN -2h (single phase TiN) and MXene electrodes to evaluate the catalytic ability

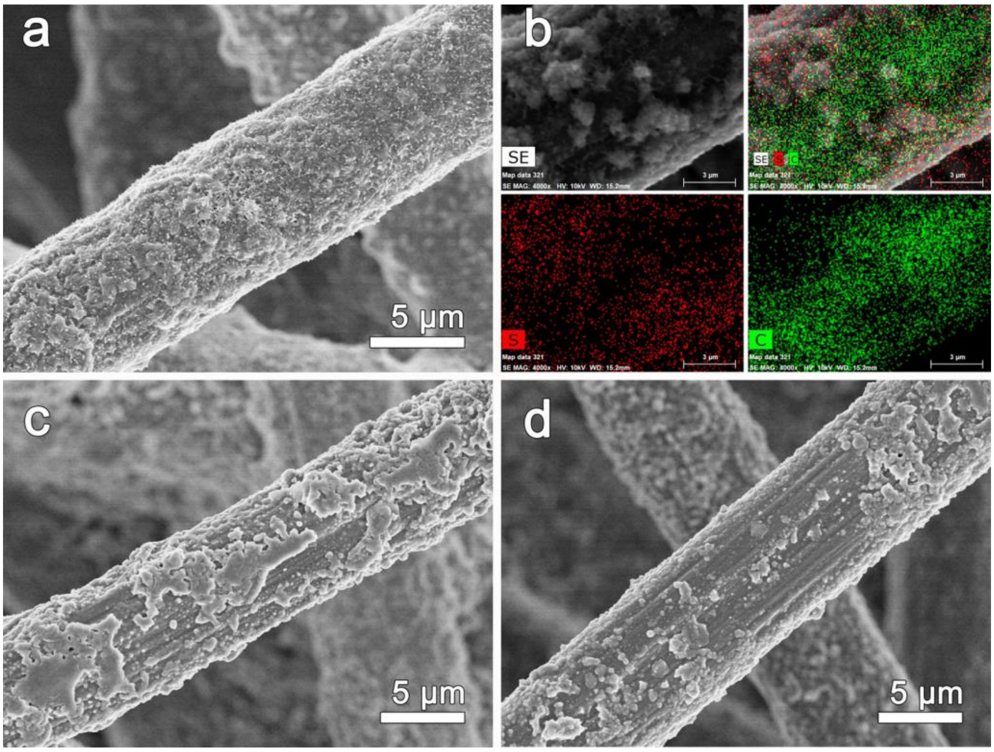

Fig. S20 SEM images showing Li_2S deposition on (**a**) MX-TiN, (**c**) MX-TiO_2 and (**d**) MXene electrodes, (**b**) EDS mapping of Li_2S nucleation on MX-TiN electrode

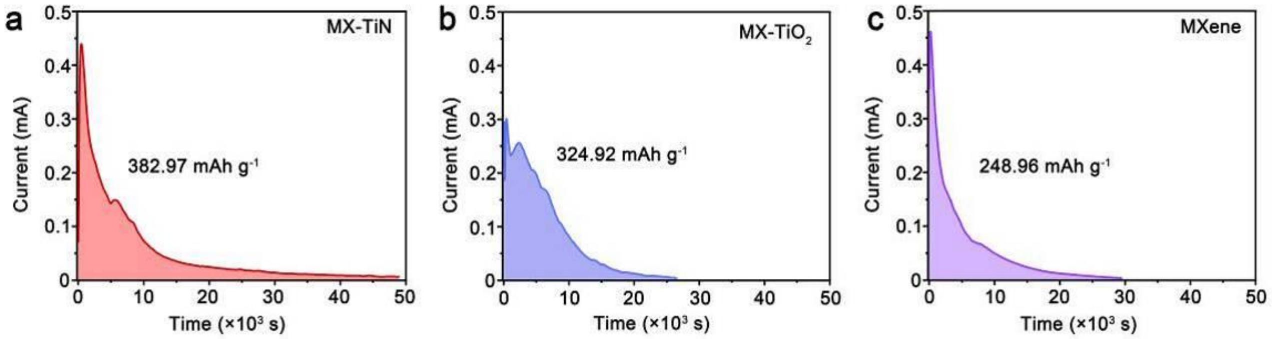

Fig. S21 Potentiostatic charge profile of Li_2S decomposition on (**a**) MX-TiN, (**b**) MX-TiO_2, and (**c**) MXene

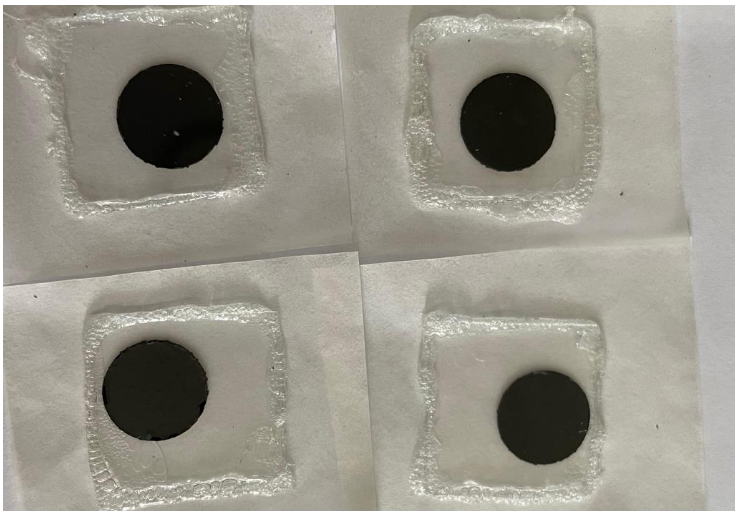

Fig. S22 Digital photo of S/MX-TiN cathodes at different potentials encapsulated in glass

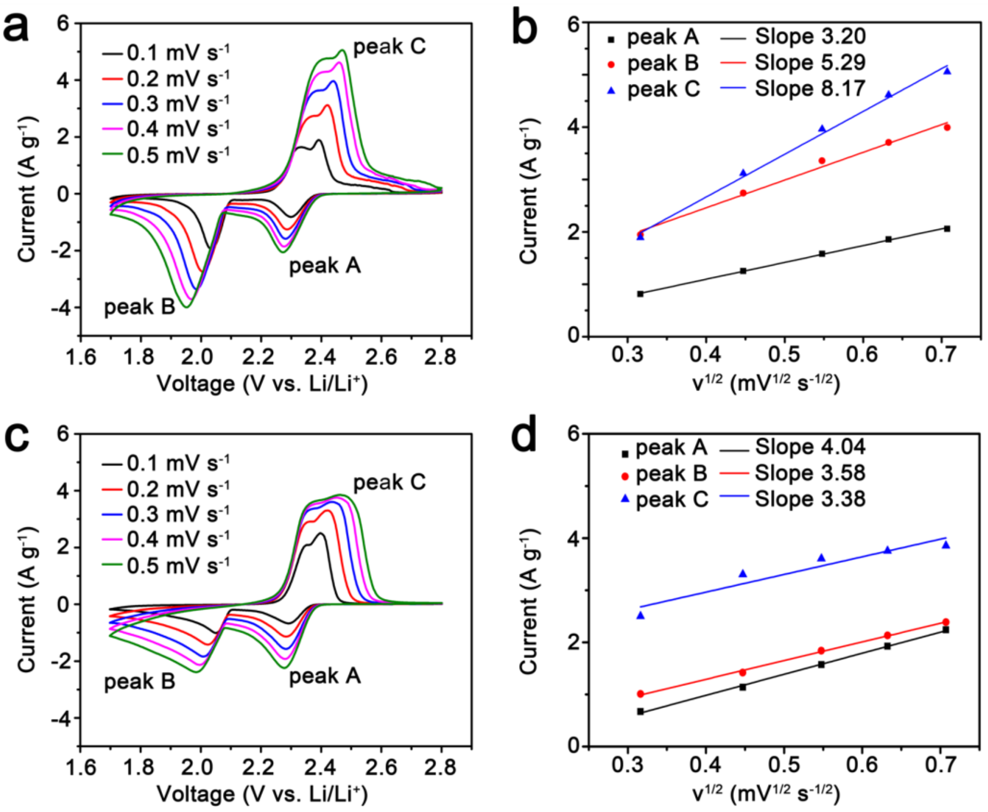

Fig. S23 CV curves with different scan rates of (**a**) MX-TiO_2, (**c**) MXene cathodes at different scan rates. Corresponding I-$v^{0.5}$ linear fitting of (**c**) MX-TiO2 and (**d**) MXene cathodes

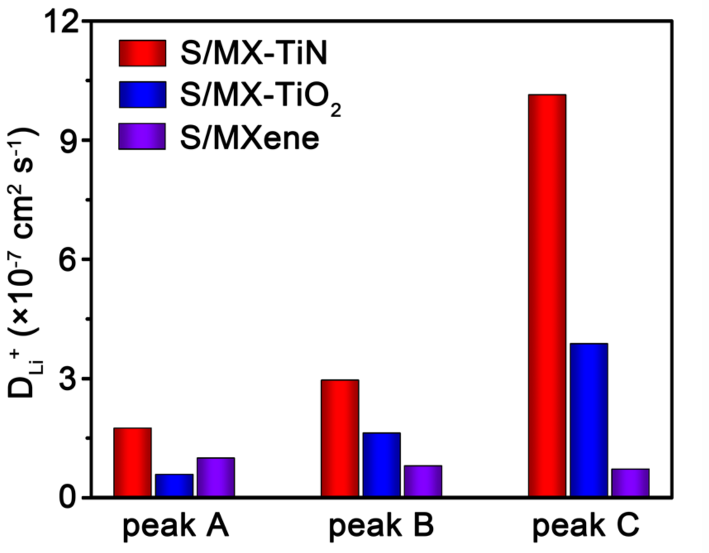

Fig. S24 Lithium-ion diffusion coefficient (D_{Li}^{+}) of S/MX-TiN, S/MX-TiO_2 and S/MXene cathodes

Randles-Sevcik equation, $I = 2.686\times10^5 n^{1.5} A D_{Li}{}^{+} C v^{0.5}$

Where F is the faraday-constant (F = 96500 C mol^{-1}), n stands for the number of transferred electrons, T represents the degree Kelvin (K) of testing environment, R is universal gas constant (R = 8.314 J (mol K)$^{-1}$), A is the area of electrode (cm^{-2}), C represents the concentration of shuttle ion (mol cm^{-3}, it is 1 for Li^+), $D_{Li}{}^{+}$ is the diffusion coefficient of Li^+ and v is the scanning rate (mV s^{-1}). The diffusion coefficient of Li^+ is easily to be work out according to the fitting slopes of I and $v^{0.5}$.

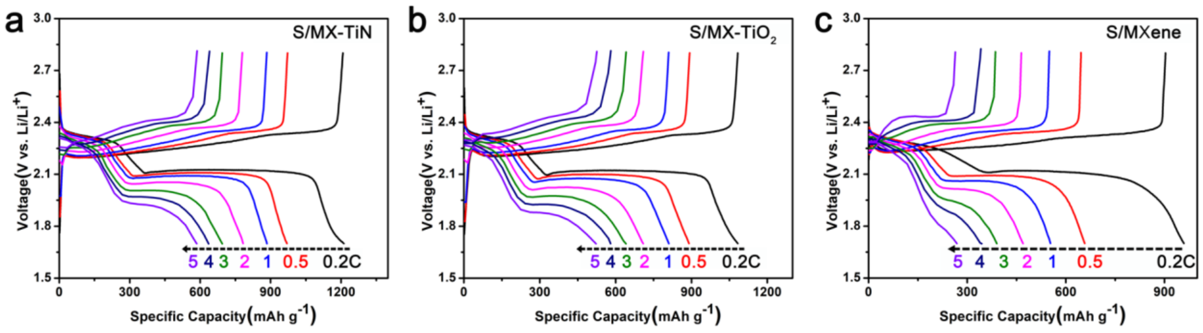

Fig. S25 Galvanostatic discharge-charge profiles of Li-S batteries with (**a**) S/MX-TiN, (**b**) S/MX-TiO_2 and (**c**) S/MXene cathodes at different current densities.

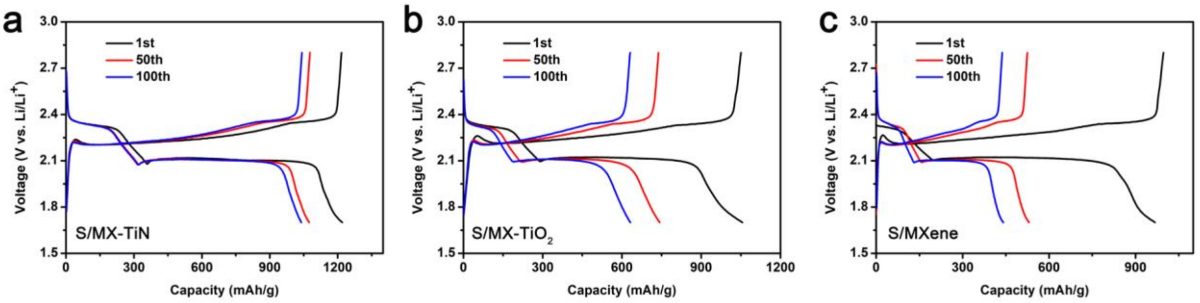

Fig. S26 Galvanostatic discharge-charge profiles of Li-S batteries with (**a**) S/MX-TiN, (**b**) S/MX-TiO_2 and (**c**) S/MXene cathodes at 0.2 C during different cycles

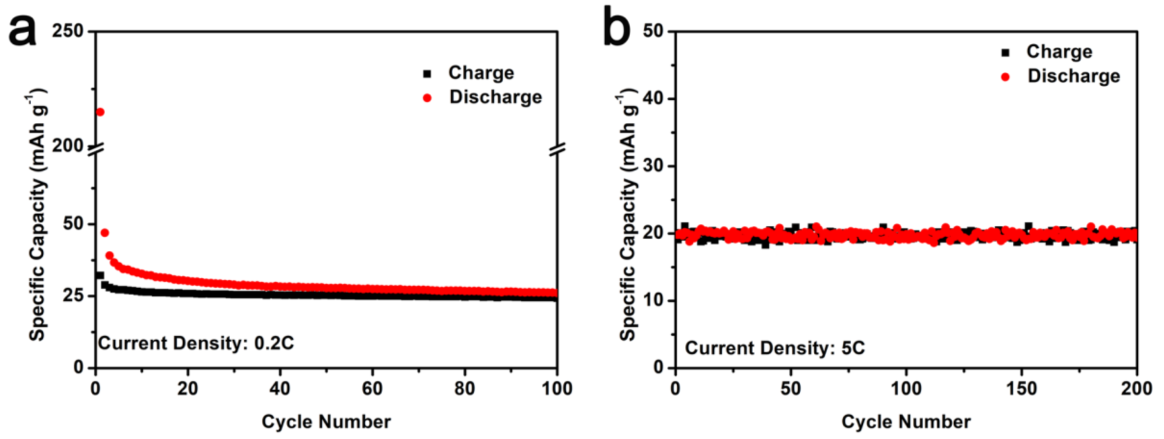

Fig. S27 Galvanostatic discharge-charge performance of pure MX-TiN without sulfur loading. Specific capacity of pure MX-TiN of (**a**) 0.2C and (**b**) 5C over a voltage range of 1.7 -2.8 V. (1C = 1672 mA g^{-1})

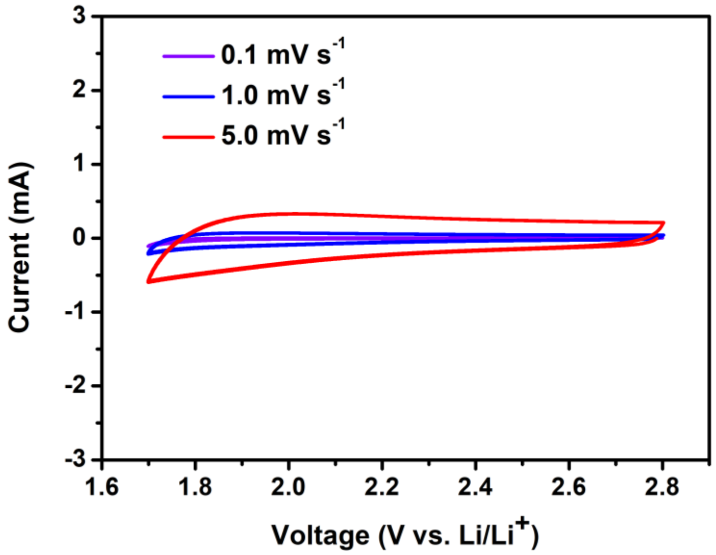

Fig. S28 CV profiles of pure MX-TiN over a voltage range of 1.7 -2.8 V at different scan rates

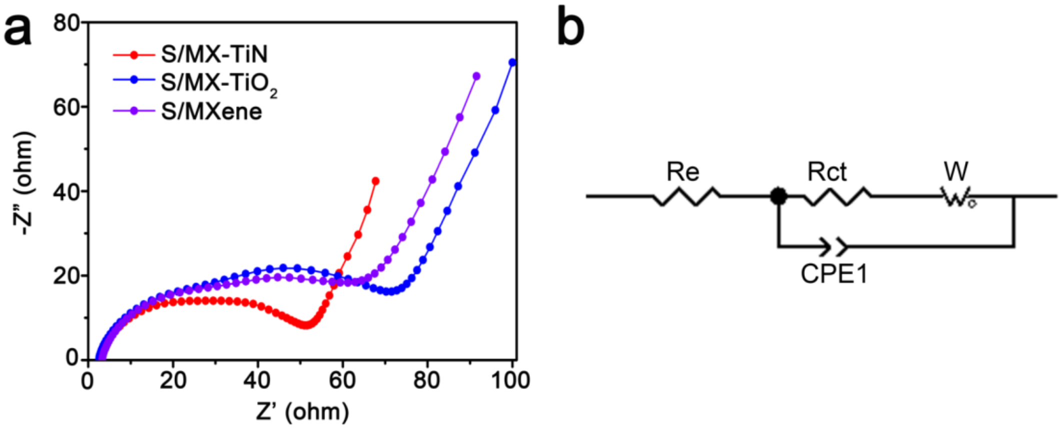

Fig. S29 (**a**) EIS of fresh cells with different cathodes, (**b**) corresponding equivalent circuit

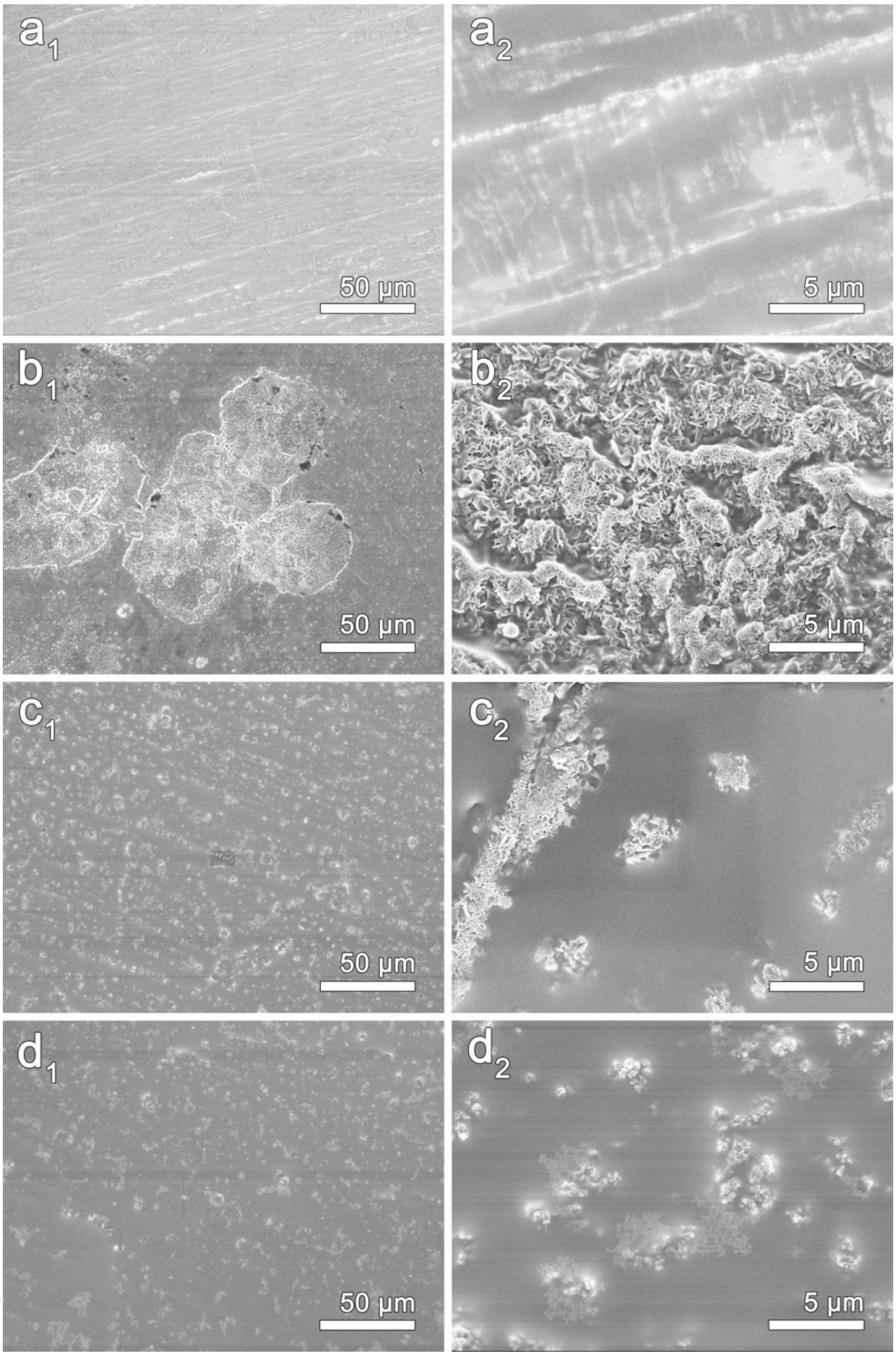

Fig. S30 (**a$_1$, a$_2$**) SEM of fresh lithium metal, SEM images of corresponding lithium surface for (**b$_1$, b$_2$**) S/MXene (**c$_1$, c$_2$**) S/MX-TiO_2 and (**d$_1$, d$_2$**) S/MX-TiN cells after 50 cycles

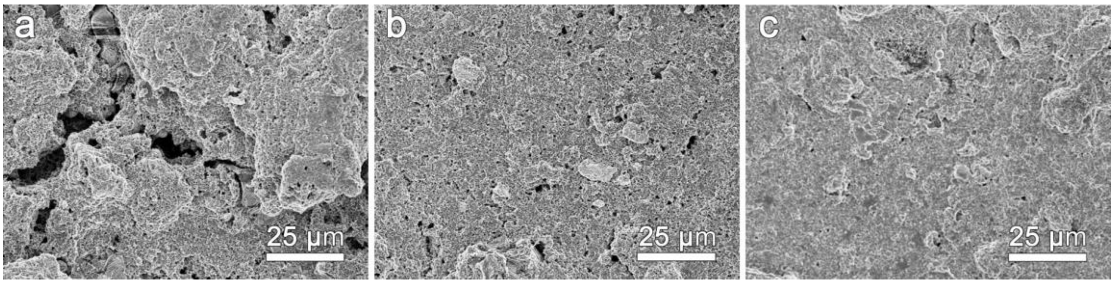

Fig. S31 SEM images for (**a**) S/MXene, (**b**) S/MX-TiO_2 and (**c**) S/MX-TiN cathodes after 50 cycles

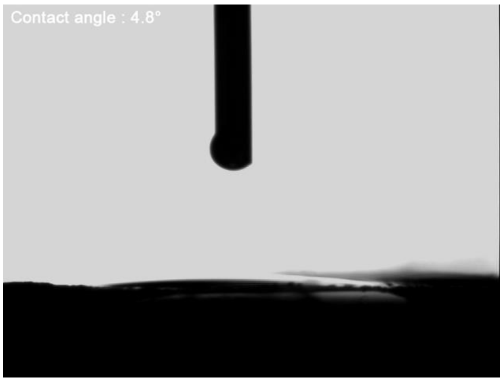

Fig. S32 The contact angle of electrolyte with S/MX-TiN cathode

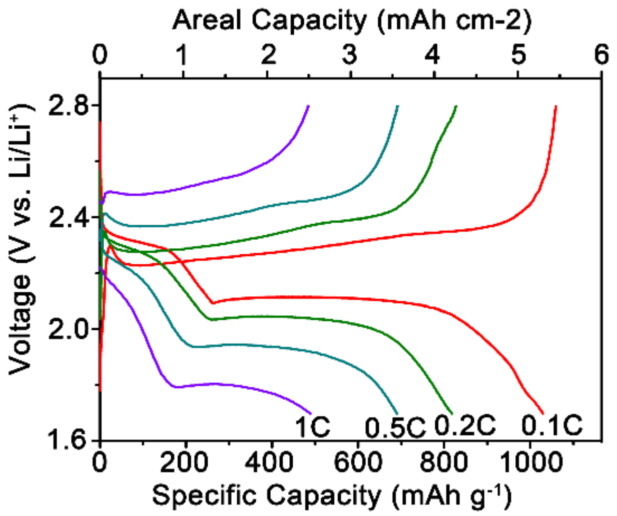

Fig. S33 Discharge-charge profiles of S/MX-TiN with a sulfur loading of 5.15 mg cm^{-2} with the E/S ratio of 11.61 μL mg^{-1}

Table S1 Atomic ratio of MX-TiN

Sample	Method	Ti	N	C	O	Ti/N
MX-TiN	EDS	24.93	11.87	42.78	20.41	2.10
	XPS	23.22	11.50	27.90	37.79	2.02

Note that elements C and O are susceptible to environmental factors, so only the atomic ratio of Ti to N is considered. (The background of EDS test is conductive carbon paper, thus the content of C element is so high.)

Table S2 BET specific surface area compared with other reported MXene-based heterostructured materials

Materials	Specific Surface Area (m^2 g^{-1})	Refs.
Sb/Na-$Ti_3C_2T_x$	44.9	[S1]
S-TC-1	50.16	[S2]
$Fe_{x-1}Se_x$/MXene/FCR	62.31	[S3]
TiO_2-$Ti_3C_2T_x$	64	[S4]
Ti_3C_2@iCON	66	[S5]
CoS NP@NHC	80	[S6]
Fe_3O_4/MXene-7	99.3	[S7]
Te-SnS_2@MXene	180.4	[S8]
N-MXene@MWCNT-MP	189.0	[S9]
OV-T_nQDs@PCN	198.2	[S10]
**MX-TiN**	**213.08**	**This work**

Table S3 Impedance parameters simulated from the equivalent circuits

	S/MX-TiN	S/MX-TiO_2	S/MXene
R_e	2.33	2.10	2.85
R_{ct}	45.99	69.13	61.38

Supplementary References

[S1] R. Zhao, H. Di, C. Wang, X. Hui, D. Zhao et al., Encapsulating ultrafine Sb nanoparticles in Na^+ pre-intercalated 3D porous $Ti_3C_2T_x$ MXene nanostructures for enhanced potassium storage performance. ACS Nano **14**(10), 13938-13951 (2020). https://doi.org/10.1021/acsnano.0c06360

[S2] J. Li, L. Han, Y. Li, J. Li, G. Zhu et al., MXene-decorated SnS_2/Sn_3S_4 hybrid as anode material for high-rate lithium-ion batteries. Chem. Eng. J. **380**, 122590 (2020). https://doi.org/10.1016/j.cej.2019.122590

[S3] J. Cao, L. Wang, D. Li, Z. Yuan, H. Xu et al., $Ti_3C_2T_x$ MXene conductive layers supported bio-derived $Fe_{x-1}Se_x$/MXene/carbonaceous nanoribbons for high-performance half/full sodium-ion and potassium-ion batteries. Adv. Mater. **33**(34), 2101535 (2021). https://doi.org/10.1002/adma.202101535

[S4] L. Jiao, C. Zhang, C. Geng, S. Wu, H. Li et al., Capture and catalytic conversion of polysulfides by in situ built TiO_2-MXene heterostructures for lithium-sulfur batteries. Adv. Energy Mater. **9**(19), 1900219 (2019). https://doi.org/10.1002/aenm.201900219

[S5] P. Li, H. Lv, Z. Li, X. Meng, Z. Lin et al., The electrostatic attraction and catalytic effect enabled by ionic-covalent organic nanosheets on MXene for separator modification of lithium-sulfur batteries. Adv. Mater. **33**(17), 2007803 (2021). https://doi.org/10.1002/adma.202007803

[S6] L. Yao, Q. Gu, X. Yu, Three-dimensional MOFs@MXene aerogel composite derived MXene threaded hollow carbon confined CoS nanoparticles toward advanced alkali-ion batteries. ACS Nano **15**(2), 3228-3240 (2021). https://doi.org/10.1021/acsnano.0c09898

[S7] P. Zhang, N. Sun, R.A. Soomro, S. Yue, Q. Zhu et al., Interface-engineered Fe_3O_4/MXene heterostructures for enhanced lithium-ion storage. ACS Appl. Energy Mater. **4**(10), 11844-11853 (2021). https://doi.org/10.1021/acsaem.1c02649

[S8] H. Sun, Y. Zhang, X. Xu, J. Zhou, F. Yang et al., Strongly coupled Te-SnS_2/MXene superstructure with self-autoadjustable function for fast and stable potassium ion storage. J. Energy Chem. **61**, 416-424 (2021). https://doi.org/10.1016/j.jechem.2021.02.001

[S9] W. Bao, R. Wang, C. Qian, Z. Zhang, R. Wu et al., Heteroatom-doped $Ti_3C_2T_x$ MXene microspheres enable strong adsorption of sodium polysulfides for long-life room-temperature sodium-sulfur batteries. ACS Nano **15**(10), 16207-16217 (2021). https://doi.org/10.1021/acsnano.1c05193

[S10] H. Zhang, L. Yang, P. Zhang, C. Lu, D. Sha et al., MXene-derived Ti_nO_{2n-1} quantum dots distributed on porous carbon nanosheets for stable and long-life Li-S batteries: enhanced polysulfide mediation via defect engineering. Adv. Mater. **33**(21), 2008447 (2021). https://doi.org/10.1002/adma.202008447
